# The TOR pathway modulates cytoophidium formation in *Schizosaccharomyces pombe*

**DOI:** 10.1074/jbc.RA119.009913

**Published:** 2019-08-19

**Authors:** Christos Andreadis, Lydia Hulme, Katherine Wensley, Ji-Long Liu

**Affiliations:** ‡ School of Life Sciences and Technology, ShanghaiTech University, 201210 Shanghai, China; § MRC Functional Genomics Unit, Department of Physiology, Anatomy and Genetics, University of Oxford, Oxford OX1 3PT, United Kingdom

**Keywords:** Schizosaccharomyces pombe, target of rapamycin (TOR), nucleotide, pyrimidine, yeast, yeast genetics, TOR complex (TORC), subcellular organelle, signal transduction, metabolic regulation, CTP synthase, cytoophidium, fission yeast, kinase signaling, protein filamentation

## Abstract

CTP synthase (CTPS) has been demonstrated to form evolutionarily-conserved filamentous structures termed cytoophidia whose exact cellular functions remain unclear, but they may play a role in intracellular compartmentalization. We have previously shown that the mammalian target of rapamycin complex 1 (mTORC1)–S6K1 pathway mediates cytoophidium assembly in mammalian cells. Here, using the fission yeast *Schizosaccharomyces pombe* as a model of a unicellular eukaryote, we demonstrate that the target of rapamycin (TOR)-signaling pathway regulates cytoophidium formation (from the *S. pombe* CTPS ortholog Cts1) also in *S. pombe*. Conducting a systematic analysis of all viable single TOR subunit–knockout mutants and of several major downstream effector proteins, we found that Cts1 cytoophidia are significantly shortened and often dissociate when TOR is defective. We also found that the activities of the downstream effector kinases of the TORC1 pathway, Sck1, Sck2, and Psk1 S6, as well as of the S6K/AGC kinase Gad8, the major downstream effector kinase of the TORC2 pathway, are necessary for proper cytoophidium filament formation. Interestingly, we observed that the Crf1 transcriptional corepressor for ribosomal genes is a strong effector of Cts1 filamentation. Our findings connect TOR signaling, a major pathway required for cell growth, with the compartmentalization of the essential nucleotide synthesis enzyme CTPS, and we uncover differences in the regulation of its filamentation among higher multicellular and unicellular eukaryotic systems.

## Introduction

Nucleotides are required for many vital processes in the cell, such as replication, transcription, and DNA repair. Consequently, NTP homeostasis is important for cell survival. CTP production is achieved through either a salvage pathway or by *de novo* synthesis (1, 2). The essential metabolic enzyme CTP synthase (CTPS)^2^ is critical for the *de novo* pathway and catalyzes the ATP-dependent transfer of nitrogen from glutamine to UTP, forming glutamate and CTP (3, 4). An inability to regulate CTP pools has been associated with a variety of malignancies, while CTPS is among the most overexpressed proteins in multiple human cancers. Thus, CTPS is an attractive target for the development of anti-cancer drugs (5–14).

Interestingly, CTPS has been found to be assembled in filamentary structures termed cytoophidia, first in *Drosophila* (15), bacteria (16), and budding yeast (17), and soon afterward in mammalian cells (18) and fission yeast (19). The formation of cytoophidia across diverse species suggests that these filaments possess an important evolutionarily-conserved biological function (20). The role of cytoophidia is enigmatic, although several physiological functions have been proposed, ranging from cytoskeleton-like functions (16) to metabolic control and buffering (21–24), to protein stabilization, stress coping (21), cell proliferation (25, 26), and intracellular transport (20, 27, 28). Recently, it has been shown that CTPS filaments may contain an inactive or active form of the enzyme in different organisms (29). However, the formation and regulation of these filaments remain elusive (15, 16, 18, 20, 30–32).

The highly-conserved target of rapamycin (TOR) serine/threonine pathway is essential for the regulation of cell growth and the response to nutrient deprivation and to further environmental cues (33–35). Dysregulation of TOR has been reported to result in diseases such as cancer, immune dysfunction, diabetes, obesity, and autism (36–41).

TOR pathway consists of two distinct, evolutionarily-conserved TOR kinase complexes, TORC1 and TORC2. In mammalian cells, there is a single TOR protein kinase (mTOR) in both complexes, while there are two in *Schizosaccharomyces pombe* and *Saccharomyces cerevisiae*. In *S. pombe*, Tor1 and Tor2 kinases are contained in TORC2 and TORC1 complexes, respectively (42, 43). Each of the two complexes is composed of four evolutionarily-conserved regulatory subunits. Pop3 is a common member of both complexes; Mip1, Tco89, and Toc1 are components of TORC1, and Ste20, Sin1, and Bit61 are specific to TORC2 (42–44). Mip1 and Ste20 are orthologs of the TORC1- and TORC2-specific human proteins Raptor and Rictor, respectively (43, 45). Notably, TORC1 and TORC2 sub-complexes have distinct roles. TORC1 is essential for vegetative growth, responsive to the availability of nitrogen and amino acids, facilitates anabolic processes, and inhibits catabolic ones, while it regulates the switch between growth and sexual development in nitrogen starvation (45–48). TORC2 is not essential for vegetative growth but is responsive to glucose availability, is required for cell survival under a variety of stresses (35, 49), and has been implicated in multiple processes, such as the timing of mitosis (46), gene silencing, telomere length maintenance (49), and amino acid uptake (50).

We have recently shown that the mTORC1–S6K1 pathway mediates cytoophidium assembly in mammalian cells and that mTOR is required for cytoophidium assembly in *Drosophila* (51). Here, we tested the universality of this type of regulation in lower eukaryotes, using the unicellular eukaryotic organism *S. pombe*. Indeed, our results showed that the TOR protein kinase–signaling pathway affects Cts1 cytoophidia formation in fission yeast. In contrast to mammalian systems, not only TORC1 but both TORC1 and TORC2 sub-complexes participate in the regulation of Cts1 cytoophidia formation. Furthermore, we have identified specific TORC1 and TORC2 effector kinases, as well as the Crf1 transcriptional corepressor, as mediators of the regulation of Cts1 filamentation. Our study shows that a similar to mammalian systems regulatory mechanism for Cts1 cytoophidia formation exists in the lower eukaryotic organism *S. pombe*, and it highlights the different ways by which this regulation is mediated.

## Results

### Cts1 cytoophidium formation is compromised under TOR inhibition

**Figure 1.**
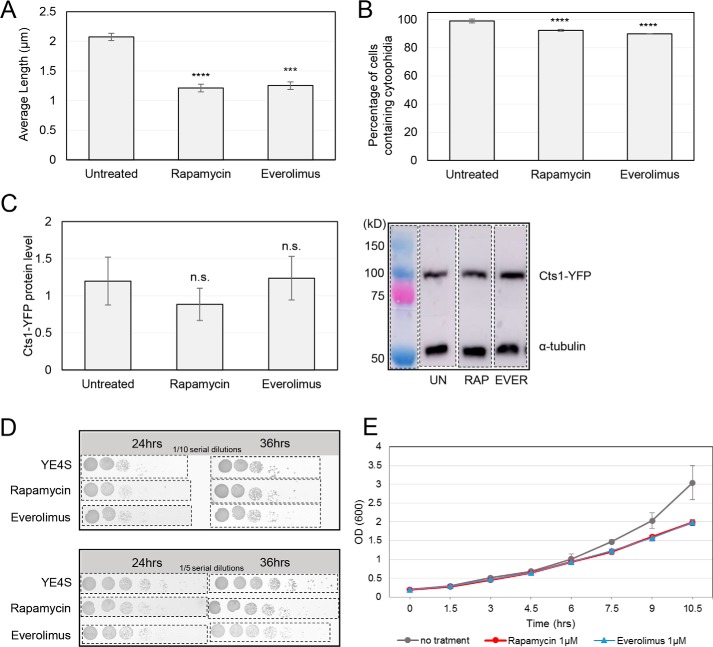
**TOR inhibitors attenuate cytoophidia formation.** Cells of the Cts1–YFP background were grown in rich medium until exponential phase, followed by a 4-h incubation with TORC1 inhibitors rapamycin (1 μm) and everolimus (1 μm). The cells were subsequently fixed, and Cts1–YFP protein was observed by fluorescent microscopy. *A,* the average length of cytoplasmic cytoophidia in cells grown under TOR inhibition was calculated and plotted along with the average length of cytoplasmic cytoophidia in cells grown under no treatment. *B,* the quantification of the cells with visible cytoplasmic cytoophidia is plotted and expressed as percentage of cells containing cytoophidia under no treatment and under growth upon TORC1 inhibitors. *Error bars* show the mean ± S.D.: as calculated from three independent experiments (>400 cells were manually counted per strain per trial; ****, *p* < 0.0001; ***, *p* < 0.001). *C,* Western blot analysis of Cts1–YFP cultures after treatment with rapamycin and everolimus, as described above. Proteins were extracted from equal number of cells, and samples were analyzed by SDS-PAGE. The Cts1–YFP protein levels under no treatment and following treatment with rapamycin and everolimus were plotted (*left*) after normalization over α-tubulin levels. A representative image of the Western blot analysis is shown on the *right,* along with the α-tubulin levels. *Error bars* show the mean ± S.D.: as calculated from three independent experiments (*n.s.,* not significant). *Dotted lines* indicate the areas of the membrane that have been cut out. *D,* cells of Cts1–YFP background were grown in YE4S until reaching an OD_600_ ∼1. Seven serial dilutions (1:10, *top panel*; 1:5, *bottom panel*) were spotted on plain YE4S agar plates (*1st row*), and on YE4S plates containing 1 μm rapamycin (*2nd row*), or 1 μm everolimus (*3rd row*). Photos of the plates were taken after 24 and 36 h of incubation at 30 °C. *Dotted lines* indicate the photographed areas that have been cut out. *E,* early log phase cells of Cts1–YFP background were cultured in YE4S and in YE4S containing rapamycin or everolimus at a final concentration of 1 μm, and growth curves were constructed after monitoring the optical density for a period of ∼10 h. The experiment was repeated in triplicate, and *error bars* show the mean ± S.D.: as calculated after three biological repeats. The slower growth after treatment with rapamycin and everolimus is significant for time points 7.5, 9, and 10.5 (*p* < 0.05).

Drug inhibition of TOR signaling has been shown to reduce cytoophidium formation in mammalian cells (51). To test whether the same is true for fission yeast, we utilized everolimus and rapamycin, two well-established allosteric inhibitors of TORC1 pathway. Both drugs exert their effects by binding to the propyl isomerase FKBP12 (FK506-binding protein 1A, 12 kDa) and forming an inhibitory complex that, in turn, binds to TORC1 with high affinity (52, 53). To study the changes in the formation of cytoophidia, we used a strain that expresses a Cts1–YFP hybrid protein (19). As in previous studies of cytoophidia in yeasts (17, 19, 23, 54, 55), endogenous tagging was preferred over immunostaining because the use of the latter was hindered by the enzymatic treatment necessary to render the fission yeast cell wall permeable to antibodies, a procedure that leads to dissociation of the sensitive structure of cytoophidia (Fig. S1). The cells were grown in rich medium until exponential phase and treated with TORC1 inhibitors rapamycin and everolimus. The average length of cytoophidia was significantly reduced from 2.075 μm (S.D.: ±0.063 μm) in untreated cells to 1.21 μm (S.D.: ±0.064 μm) and 1.25 μm (S.D.: ±0.062 μm) after treatment with rapamycin and everolimus, a reduction of 41.8% (*p* < 0.0001) and 40% (*p* < 0.001), respectively (Fig. 1*A*). The percentage of cells containing cytoophidia showed a small, yet significant (*p* < 0.0001), reduction (Fig. 1*B*). The Cts1 protein levels remained relatively unchanged after treatment with TORC1 inhibitors (Fig. 1*C*). The growth rate of the cells after addition of rapamycin and everolimus TOR inhibitors in the growth media is mildly reduced (Fig. 1, *D* and *E*). In agreement with our data, it has been demonstrated that rapamycin only mildly reduces the growth rate in fission yeast cells, whereas TORC1 activation is significantly inhibited (56–58), as is in mammalian cells treated with rapamycin (51).

Overall, as observed previously in mammalian cells, pharmacological inhibition of the TOR pathway affects cytoophidia formation, but in the case of *S. pombe*, this is manifested predominantly by reduction of the length of Cts1 filaments, rather than by changes in the number of cells containing cytoophidia.

### Knockout mutants of TORC2 and TORC1 components disrupt cytoophidia formation

TOR protein kinase complex consists of TORC1 and TORC2 sub-complexes, each of which is composed of five subunits, while the TOR pathway signal is transduced via several downstream effectors to its targets. To gain a better understanding of the Cts1 filamentation regulation by TOR signaling, we aimed to evaluate the effect each component has on cytoophidia formation. To do that, we constructed a series of all viable knockout mutants of both TOR sub-complexes and studied the effect on CTPS filaments.

**Figure 2.**
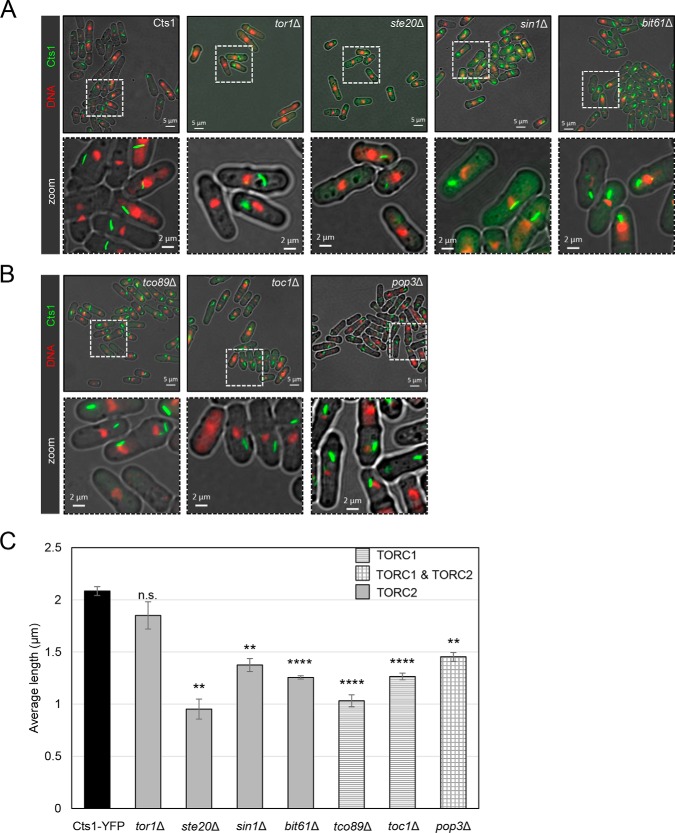
**Reduced cytoophidium length is observed in TORC2 and TORC1 knockout mutants.** Cells were grown in rich medium until exponential phase, followed by fixation and observation of Cts1–YFP protein by fluorescence microscopy. *A,* representative images of control and TORC2 mutant strains (*tor1*Δ, *ste20*Δ, *sin1*Δ, and *bit61*Δ) are shown (*top row*), along with details of the cytoophidium morphology in a magnified area (*bottom row*). *B,* representative images of TORC1 mutant strains (*tco89*Δ and *toc1*Δ) and *pop3*Δ (TORC1 and TORC2 sub-complex) are shown in the *top row*, and the localized magnified areas are shown in the *bottom row. C,* the average length of cytoplasmic cytoophidia was calculated in each TOR mutant and plotted along with the average length of cytoophidia in the Cts1–YFP strain. The different pattern indicates participation in TORC1, TORC2, or both complexes, as shown. *Error bars* show the mean ± S.D.: as calculated from three independent experiments (the length of cytoophidia in >400 cells was manually measured per strain per trial; ****, *p* < 0.0001; **, *p* < 0.01; *n.s.,* not significant). All TOR mutant strains were constructed in a Cts1–YFP background.

More specifically, subunits of the TORC2 complex were deleted in a Cts1–YFP background, in order to generate viable haploid single TOR mutant strains expressing the YFP-tagged form of CTPS. This included the major TORC2 kinase (Tor1) and the TORC2 subunits Ste20, Bit61, and Sin1. Similarly, we have constructed the TORC1 deletion strains *tco89*Δ and *toc1*Δ in a Cts1–YFP background, as well as the strain in which we deleted *Pop3*, a gene that normally encodes a protein that participates in both TORC1 and TORC2 complexes. We subsequently monitored the filament formation of Cts1–YFP in TORC2 and TORC1 mutants by fluorescence microscopy (Fig. 2, *A* and *B*, respectively). The average length of cytoophidia was significantly reduced in almost all TORC1 and TORC2 knockout mutants tested (Fig. 2*C*). For the TORC2 mutants, the reduction of the average length, as compared with the Cts1–YFP (2.08 μm; S.D.: ±0.043 μm), was 54.3% in *ste20*Δ (0.95 μm; S.D.: ±0.096 μm, *p* < 0.01), 34.1% in *sin1*Δ (1.37 μm; S.D.: ±0.062 μm, *p* < 0.01), 39.8% in *bit61*Δ (1.26 μm; S.D.: ±0.015 μm, *p* < 0.0001), and 11.2% in *tor1*Δ (1.85 μm; S.D.: ±0.13 μm, *p* > 0.05). Similarly, for the TORC1 mutants, the average length of cytoophidia was reduced by 50.6% in *tco89*Δ cells (1.03 μm; S.D.: ±0.057 μm, *p* < 0.0001) and by 39.3% in *toc1*Δ cells (1.26 μm; S.D.: ±0.033 μm, *p* < 0.0001). For the *pop3*Δ mutant strains, the average length of CTPS filaments was reduced by 30.3% (1.45 μm; S.D.: ±0.042 μm, *p* < 0.01).

The shift from longer to shorter cytoophidia for the TORC1 and TORC2 subunit knockout mutants is further demonstrated in Fig. 4*A*. Here, the CTPS filaments were grouped into three categories according to their length (small: L <0.8 μm; medium: 0.8 μm < L <1.6 μm; large: L >1.6 μm), and the percentage of cytoophidia distributed in each length group was plotted for the control and mutant strains. The percentage of the sum of small and medium cytoophidia was 21.1% (S.D.: ±5.28%) for the control strain, although it shifted to 94.5% (S.D.: ±3.67%) in *ste20*Δ cells (4.5-fold shift, *p* < 0.0001), 84.8% (S.D.: ±2.69%) in *tco89*Δ cells (4-fold shift, *p* < 0.001), 75.5% (S.D.: ±3.26%) in *bit61*Δ cells (3.6-fold shift, *p* < 0.001), 75.2% (S.D.: ±2.29%) in *toc1*Δ cells (3.6-fold shift, *p* < 0.001), 69.7% (S.D.: ±6.11%) in *sin1*Δ cells (3.3-fold shift, *p* < 0.01), 61.6% (S.D.: ±2.12%) in *pop3*Δ cells (2.9-fold shift, *p* < 0.001), and 42.5% (S.D.: ±3.08%) in *tor1*Δ cells (2-fold shift, *p* < 0.01).

Notably, the percentage of cells containing cytoophidia was also significantly disrupted in knockout mutants of both TORC1 and TORC2 components, as compared with the Cts1–YFP strain (99%, S.D.: ±1.41%). More specifically, the percentage of cells containing cytoophidia in TORC2 mutants *tor1*Δ (21.49%, S.D.: ±3.39%) and *ste20*Δ (72.7%, S.D.: ±6.68%) exhibited a significant cytoophidia reduction of ∼78.3 and ∼26.6%, respectively (*p* < 0.0001) (Fig. 4*C*). The *sin1*Δ (89.7%, S.D.: ±4.01%) cells showed a small (∼9.4%) but significant (*p* < 0.01) reduction in cytoophidia formation, as did *bit61*Δ (93.5%, S.D.: ±2.35%) cells (∼9.4%, *p* < 0.01) (Fig. 4*C*). Regarding TORC1 mutants, fluorescence microscopy showed a significant 27.6% reduction in cytoophidia formation in the *tco89*Δ (71.7%, S.D.: ±11.09%) strain compared with the Cts1–YFP cells (*p* < 0.001) (Fig. 4*C*). Note that the reduction of the average CTPS filament length is a more common effect following the disruption of TORC1 or TORC2 signaling, as all single mutants of TOR components exhibit this phenotype (Fig. 4*A*). In contrast, the reduction in the percentage of cells containing cytoophidia is a phenotype present in most but not all TORC2 and TORC1 mutants, for example, it is not significant for *toc1*Δ and *pop3*Δ mutants. Additionally, in *tor1*Δ mutants the percentage of cells containing cytoophidia is strongly reduced, while the average length only mildly reduced.

Overall, our results show that cytoophidia formation is compromised when individual subunits of either TORC1 or TORC2 are knocked out, suggesting that even a partially functional TOR-signaling pathway can significantly affect CTPS filamentation. Furthermore, in contrast to mammalian systems, in which only TORC1 affects the regulation of CTPS compartmentation, in *S. pombe* both TORC1 and TORC2 subcomplexes seem to be involved.

### Downstream effectors of TORC2 and TORC1 complexes interfere with cytoophidium formation

**Figure 3.**
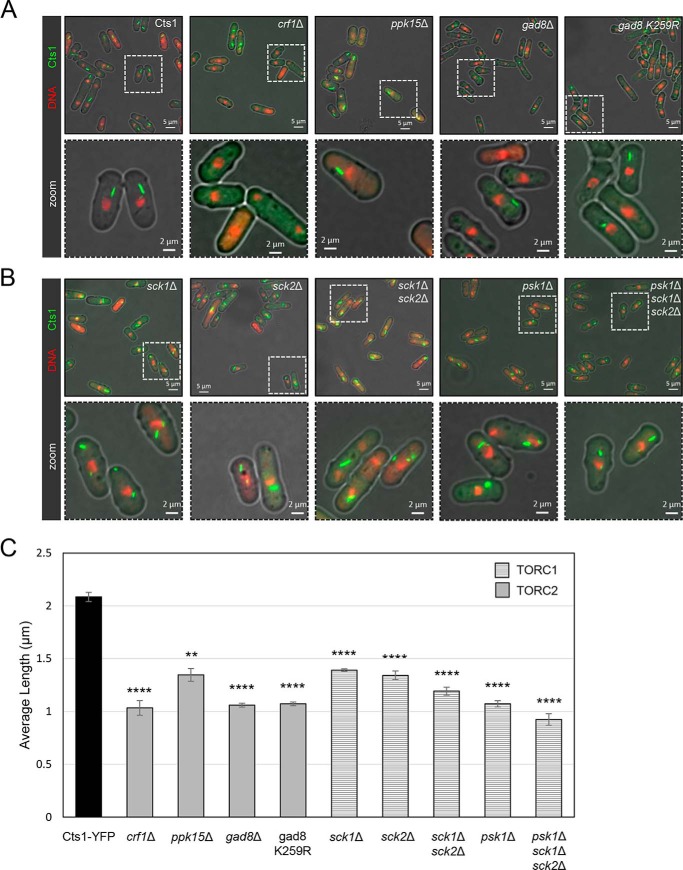
**Cytoophidium length is controlled by S6- and AGC kinase-mediated pathways downstream of TORC2 and TORC1.** Cells were grown in rich medium until exponential phase, followed by fixation and observation of Cts1–YFP protein by fluorescence microscopy. *A,* representative images of control and mutant strains of TORC2 effectors (*crf1*Δ, *ppk15*Δ, *gad8*Δ, and gad8 K259R) are shown (*top row*), along with details of the cytoophidium morphology in a magnified area (*bottom row*). *B,* representative images of mutant strains of TORC1 effectors (*sck1*Δ, *sck2*Δ, *sck1*Δ *sck2*Δ, *psk1*Δ, *psk1*Δ *sck1*Δ *sck2*Δ) (*top row*) and localized magnified area (*bottom row*). *C,* the average length of cytoplasmic cytoophidia was calculated in each TOR effector mutant and plotted along with the average length of cytoophidia in the Cts1–YFP strain. The different pattern indicates participation in TORC1 or TORC2 complexes, as indicated. *Error bars* show the mean ± S.D.: as calculated from three independent experiments (the length of cytoophidia in >400 cells was manually measured per strain per trial; ****, *p* < 0.0001; **, *p* < 0.01). All TOR mutant strains were constructed in a Cts1–YFP background.

In mammalian systems, it has been shown that CTPS filamentation is mediated by mTORC1–S6K1-signaling pathway (51). Along with establishing the effects of the absence of single TORC1 and TORC2 subunits on Cts1 filaments in *S. pombe*, we explored whether the TOR downstream regulatory phosphorylation cascade system and/or other downstream effectors could mediate the formation of cytoophidia in fission yeast. To address this, we constructed a series of knockout mutants of TOR downstream effectors in a Cts1–YFP background and monitored the changes on Cts1 filaments (Fig. 3, *A* and *B*).

**Figure 4.**
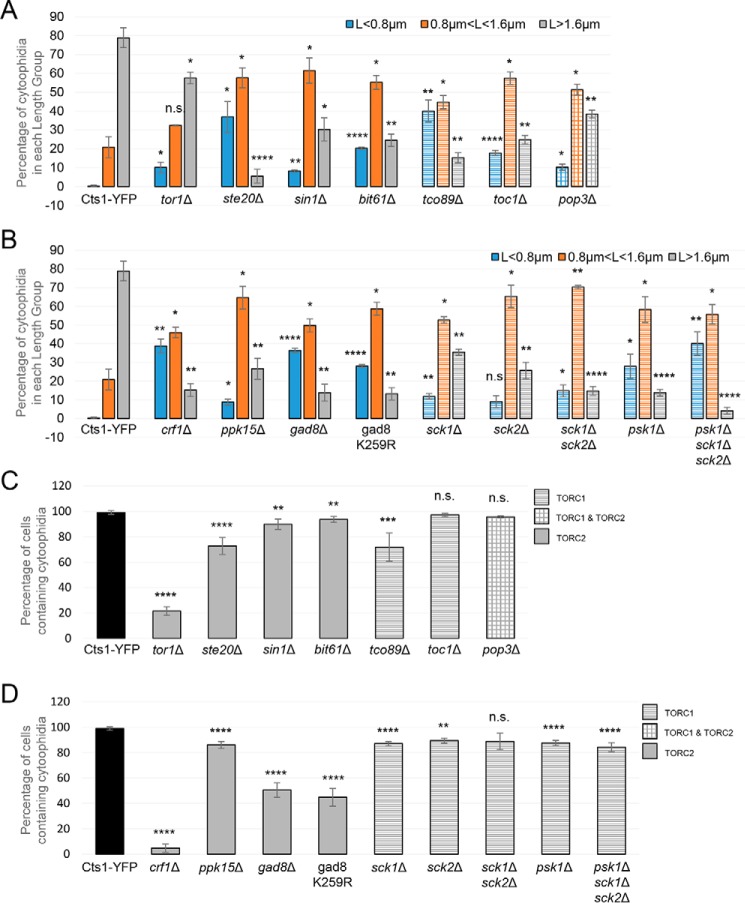
**TOR mutants show a shift to forming shorter Cts1 filaments and an overall reduction of cytoophidia-containing cells.** Exponentially growing cells in YE4S were fixed and observed by fluorescence microscopy. *A,* the average length of cytoplasmic cytoophidia was calculated in each TOR subunit mutant strain as in Figs. 2*C* and 3*C*, and cytoophidia were grouped into three categories based on their length (*L*), as shown. The distribution of cytoophidia, expressed as average percentage of cytoophidia in each category, is plotted for the mutants and the Cts1–YFP control strain. Different color coding is used to distinguish between the three categories as shown, and the different pattern indicates participation in TORC1 (*tco89*Δ and *toc1*Δ) (*horizontal lines*), TORC2 (*tor1*Δ, *ste20*Δ, *sin1*Δ, and *bit61*Δ) (*full*), or both TORC1 and TORC2 (*pop3*Δ) (*rectangles*). *B,* the same analysis as in *A* was performed for downstream effector mutants of TORC1 (*sck1*Δ, *sck2*Δ, *sck1*Δ *sck2*Δ, *psk1*Δ, and *psk1*Δ *sck1*Δ *sck2*Δ) and of TORC2 (*crf1*Δ, *ppk15*Δ, *gad8*Δ, and gad8 K259R). *C*, the quantification of the cells with visible cytoophidia is plotted for the knockout mutants of TORC2 and TORC1 subunits, expressed as percentage of cells containing cytoophidia in each of the strains tested. The different pattern indicates participation in TORC1, TORC2, or both complexes, as shown. *D,* the same analysis as in *C* was performed for the TORC1 and TORC2 downstream effector mutants. *Error bars* show the mean ± S.D.: as calculated from three independent experiments (>400 cells or cytoophidia were manually counted per strain per trial; ****, *p* < 0.0001; ***, *p* < 0.001; **, *p* < 0.01; *, *p* < 0.05; *n.s.,* not significant). All TOR mutant strains were constructed in a Cts1–YFP background.

In *S. cerevisiae*, Crf1 functions as a transcriptional corepressor downstream of the TORC1-signaling pathway, involved in the repression of ribosomal protein gene transcription via TOR (59, 60), and as such it is likely that it also lies downstream of the TORC2 complex in fission yeast, because *S. pombe*'s TORC2 is *S. cerevisiae*'s TORC1 homolog. To study the possible effects of Crf1 in the cytoophidium formation, we constructed the *crf1*Δ deletion strain in a Cts1–YFP background and monitored the Cts1 filamentation by fluorescence microscopy (Fig. 3*A*). The average length of cytoophidia, as compared with the Cts1–YFP strain (2.08 μm; S.D.: ±0.043 μm), was reduced by 50.4% to 1.03 μm (S.D.: ±0.069 μm, *p* < 0.0001) (Fig. 3*C*), suggesting that Crf1 transcriptional corepressor action for ribosomal proteins is important for CTPS filamentation. The percentage of the sum of small and medium cytoophidia shifted from 21.1% (S.D.: ±5.28%) (control strain) to 84.8% (S.D.: ±3.45%) in *crf1*Δ cells (4-fold shift, *p* < 0.001) (Fig. 4*B*). We further observed a striking 95.3% reduction (*p* < 0.0001) in cells containing cytoophidia (from 99%, S.D.: ±1.41% in the Cts1–YFP strain to 4.69%, S.D.: ±3.16% in *crf1*Δ strain) (Fig. 4*D*), indicating that the function of Crf1 is strongly involved in the regulation of cytoophidia formation.

Ppk15 Ser/Thr protein kinase acts downstream of the TORC2 pathway, while interacting genetically with the MBF transcription factor (61, 62). We constructed a *ppk15*Δ deletion strain in a Cts1–YFP background and monitored Cts1 filamentation by fluorescence microscopy (Fig. 3*A*). The average length of cytoophidia, as compared with the Cts1–YFP strain (2.08 μm; S.D.: ±0.043 μm), was reduced by 35.5% (1.35 μm; S.D.: ±0.06 μm:, *p* < 0.01) (Fig. 3*C*), and the percentage of the sum of small and medium cytoophidia shifted from 21.1% (S.D.: ±5.28%, control strain) to 73.4% (S.D.: ±5.62%) in *ppk15*Δ cells (3.5-fold shift, *p* < 0.001) (Fig. 4*B*). Furthermore, *ppk15*Δ cells showed a small (13.1%) but significant (*p* < 0.0001) reduction in cells containing cytoophidia (from 99%, S.D.: ±1.41% in the Cts1–YFP strain to 86.1%, S.D.: ±2.69% in *ppk15*Δ strain) (Fig. 4*D*).

It is known that phosphorylation and activation of the S6K/AGC kinase Gad8 mediate the majority of TORC2 functions (63, 64). Furthermore, TORC2 regulates the phosphorylation of the ribosomal protein S6 via Gad8 (65), and Gad8 has also been implicated with the regulation of the response to DNA replication stress via the MBF transcription factor (66). To check whether Gad8 is also a mediator of Cts1 cytoophidia formation, we constructed the *gad8*Δ deletion strain in a Cts1–YFP background, and we also used the catalytically inactive gad8 K259R strain (67) to study the effects on filamentation by fluorescence microscopy (Fig. 3*A*). The average length of cytoophidia in *gad8*Δ and gad8 K259R strains was reduced compared with the control strain (2.08 μm; S.D.: ±0.043 μm) by 49.2 and 48.6%, respectively (to 1.06 μm; S.D.: ±0.019 and 1.07 μm; S.D.: ±0.018 μm, respectively, *p* < 0.0001) (Fig. 3*C*). The percentage of the sum of small and medium cytoophidia shifted from 21.1% (S.D.: ±5.28%, control strain) to 86.2% (S.D.: ±4.55%) in *gad8*Δ cells (4.1-fold shift, *p* < 0.001) and to 86.7% (S.D.: ±3.26%) in gad8 K259R cells (4.1-fold shift, *p* < 0.001) (Fig. 4*B*). Both TORC2 pathway effector kinase mutants showed a significant reduction in cells containing cytoophidia, with 49.1% reduction in *gad8*Δ (*p* < 0.0001) and 54.8% reduction in the *gad8*Δ K259R strain (*p* < 0.0001) (from 99%, S.D.: ±1.41% in the Cts1–YFP strain to 50.4%; S.D.: ±5.74% in *gad8*Δ and to 44.7%; S.D.: ±7.01% in gad8 K259R strain) (Fig. 4*D*). The significant increase in the percentage of cells devoid of cytoophidia shows that Gad8 kinase effects are important in mediating Cts1 filamentation.

Similarly to the Gad8, Crf1, and Ppk15 downstream effectors of the TORC2 complex, we also studied the effects of downstream effectors of the TORC1 complex in cytoophidia formation. In particular, we studied the effects that Psk1, Sck1, and Sck2 members of the S6 AGC kinase (68, 69) have in the regulation of Cts1 filamentation, using both single and double/triple mutants (Fig. 3*B*). The average length of cytoophidia in all TORC1 downstream effector knockout mutants was significantly reduced, compared with the control strain. More specifically, as compared with the control strain (2.08 μm; S.D.: ±0.043 μm), the reduction was 33.2% in *sck1*Δ cells (1.39 μm; S.D.: 0.011 μm, *p* < 0.0001), 35.6% in *sck2*Δ cells (1.34 μm; S.D.: 0.04 μm, *p* < 0.0001), 42.8% in *sck1*Δ *sck2*Δ cells (1.19 μm; S.D.: 0.038 μm, *p* < 0.0001), 48.6% in *psk1*Δ cells (1.07 μm; S.D.: 0.029 μm, *p* < 0.0001), and 55.7% in *psk1*Δ *sck1*Δ *sck2*Δ cells (0.92 μm; S.D.: 0.054 μm, *p* < 0.0001) (Fig. 3*C*). The percentage of the sum of small and medium cytoophidia shifted from 21.1% (S.D.: ±5.28%, control strain) to 64.5% (S.D.: ±1.65%) in *sck1*Δ cells (3.1-fold shift, *p* < 0.001), to 74.3% (S.D.: ±4.35%) in *sck2*Δ cells (3.5-fold shift, *p* < 0.001), to 85.2% (S.D.: ±2.35%) in *sck1*Δ *sck2*Δ cells (4-fold shift, *p* < 0.0001), to 86.2% (S.D.: ±1.81%) in *psk1*Δ cells (4.1-fold shift, *p* < 0.0001), and to 95.9% (S.D.: ±1.86%) in *psk1*Δ *sck1*Δ *sck2*Δ cells (4.5-fold shift, *p* < 0.0001) (Fig. 4*B*). Additionally, there was a small reduction in the cells containing cytoophidia, as compared with the Cts1–YFP strain (99%, S.D.: ±1.41%), which was ∼12% (*p* < 0.0001) in *sck1*Δ cells (87.15%; S.D.: ±1.59%), 9.6% (*p* < 0.01) in *sck2*Δ cells (89.5%; S.D.: ±1.98%), 10.3% (*p* > 0.05) in *sck1*Δ *sck2*Δ cells (88.8%; S.D.: ±6.52%), 11.4% (*p* < 0.0001) in *psk1*Δ cells (87.7%; S.D.: ±2.01%), and 14.8% (*p* < 0.0001) in *psk1*Δ *sck1*Δ *sck2*Δ cells (84.3%; S.D.: ±3.51%) (Fig. 4*D*).

Overall, our results demonstrate that deletion mutants of downstream effectors of both TORC1 and TORC2 pathways, including Crf1 transcriptional corepressor, Ppk15 Ser/Thr kinase, and a series of S6K/AGC kinases, significantly affect CTPS cytoophidia formation, suggesting that TOR signaling likely regulates the Cts1 filamentation via the same molecular pathways and proteins involved in the regulation of cell growth.

### Cts1 abundance is reduced in Gad8 and Crf1 knockout mutants

Cts1 protein abundance can affect cytoophidia formation. Reduced Cts1 expression has been shown to disassemble cytoophidia in *Drosophila* follicle cells (32) and overexpression has been shown to promote cytoophidium assembly in flies (30). Recently, a high level of CTPS after overexpression has been demonstrated to induce cytoophidia formation in developing cortical neurons in mice, causing impairment of corticogenesis (70).

**Figure 5.**
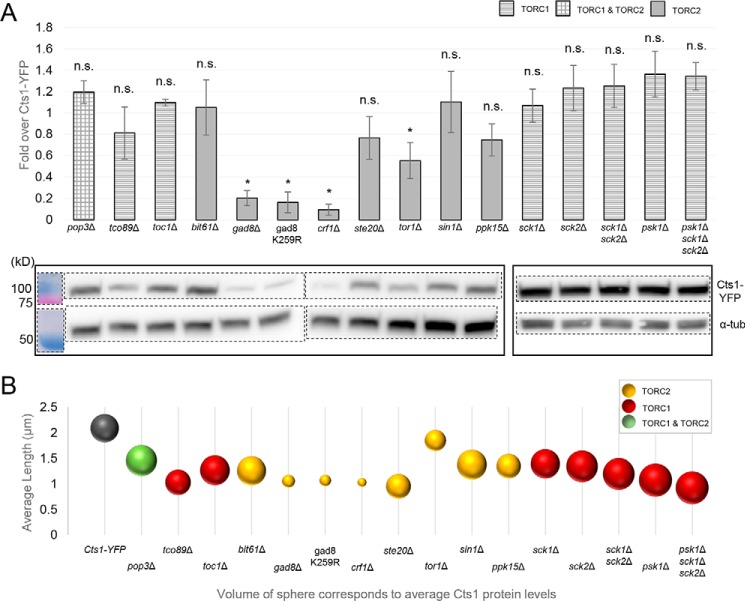
**TORC2 downstream Gad8 S6 kinase and Crf1 transcriptional corepressor knockout mutants exhibit reduced Cts1 protein levels.** *A,* cultures of knockout mutants of TORC1 and TORC2 subunits and of their downstream effector proteins were grown until exponential phase. Protein extracts were then obtained and analyzed by Western blotting. The quantification of the Cts1–YFP protein level, as obtained by three independent experiments, is presented in the *upper panel* as fold over the Cts1–YFP protein level in a nonmutated strain, after normalization over α-tubulin levels. *Error bars* show the mean ± S.D.: as calculated from three repeats (*, *p* < 0.05; *n.s.,* not significant). The *different column patterns* indicate participation in TORC1, TORC2, or both complexes, as noted. The *bottom panel* shows a representative image of the Western blot analysis of Cts1–YFP protein in the series of mutant strains, along with the α-tubulin (α-*tub*) levels. The protein bands of the last five strains are *boxed* separately, as they were electrophoresed independently. *Dotted lines* indicate the membrane areas that have been spliced. *B,* the average length of cytoophidia was calculated as described in Figs. 2*C* and 3*C* for all TOR mutants. It was then plotted against the average Cts1 protein levels contained in equal number of cells for each mutant, as measured by Western blotting. The sphere volume in each mutant corresponds to the average values of Cts1 protein, as calculated after three biological repeats. All TOR mutant strains were constructed in a Cts1–YFP background. Different coloring corresponds to participation in TORC1, TORC2, or both complexes, as indicated.

We investigated the reduction of both Cts1 cytoophidia length and abundance in TOR mutant strains by examining whether the Cts1 protein levels are altered in a TOR-defective genetic background, considering it a plausible cause of the filament reduction. Interestingly, the reduction of Cts1 protein levels in TOR mutants was significant only for the cases of the S6/AGC kinase knockout mutants *gad8*Δ and gad8 K259R, the transcriptional corepressor knockout mutant *crf1*Δ, and the knockout mutant of the major TORC2 kinase *tor1*Δ (Fig. 5*A*). For these mutant strains, the reduced abundance of Cts1 protein coincided with a reduction in the length of cytoophidia and in the percentage of cells containing cytoophidia (*p* < 0.05). However, the reduction of cytoophidia average length was not always proportional to the change of Cts1 protein levels (Fig. 5*B*). For example, the reduction of CTPS filaments' average length compared with the control strain was close to 50% in *ste20*Δ, *tco89*Δ, *crf1*Δ, *gad8*Δ, gad8 K259R, *psk1*Δ, and *psk1*Δ *sck1*Δ *sck2*Δ strains, while the CTPS protein abundance varied considerably (Fig. 5*B*). Notably, for the majority of TORC1 and TORC2 subunits and downstream effector knockout mutants, there is not a significant change in the Cts1 protein levels, compared with the control Cts1–YFP strain. Taken together, our data suggest that for three TORC2-related knockout mutants, the reduced CTPS protein levels could contribute to the reduced length and abundance of CTPS filaments; however, this is not the case for any of the TORC1 mutants or for the rest of the TORC2 knockout mutants. This could be indicative of a difference in the mode of regulation of filamentation by the two TOR complexes.

**Figure 6.**
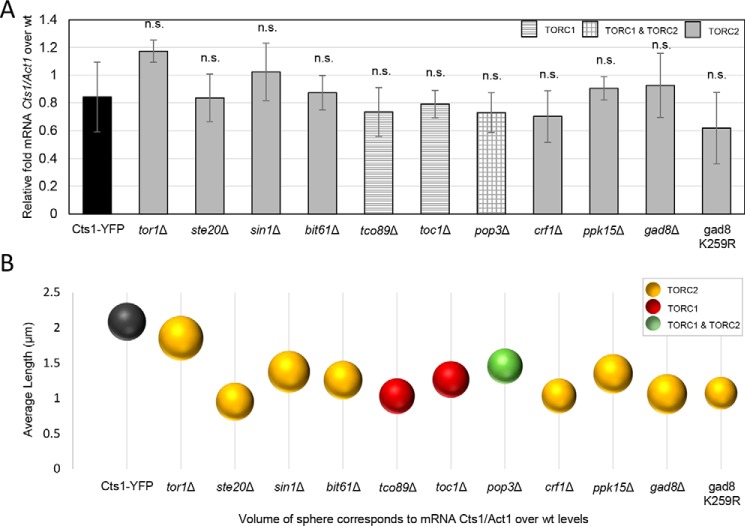
**Cts1 transcription is not affected significantly in TOR mutants.** *A, Cts1* transcript levels were calculated in TORC1 and TORC2 mutant strains by qPCR. The relative mRNA levels of *Cts1* in all strains over WT, after normalization over *Act1* mRNA levels, are presented. *Error bars* show the mean ± S.D.: as calculated from three independent experiments (*p* > 0.5; *n.s.,* not significant). *B,* the average length of cytoophidia was calculated as described in Figs. 2*C* and 3*C* for the TOR mutants. It was then plotted against the average *Cts1* mRNA levels contained in an equal number of cells for each mutant, as measured by qPCR. The sphere volume in each case corresponds to the average values of mRNA levels, as calculated after three biological repeats. All TOR mutant strains were constructed in a Cts1–YFP background.

**Figure 7.**
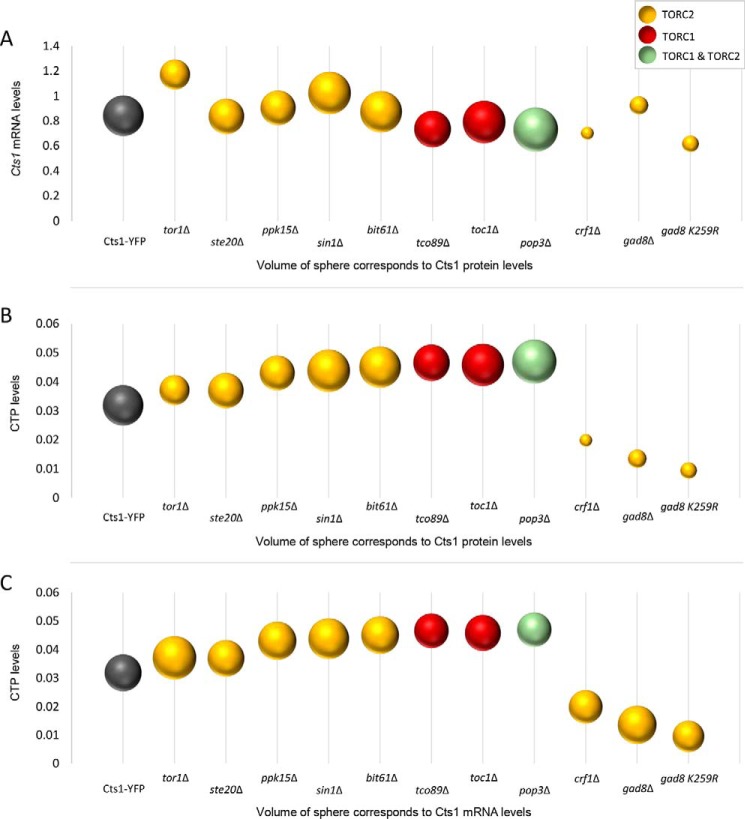
**Dynamics between Cts1 transcription, translation, and enzymatic activity.** *A,* average *Cts1* mRNA levels are plotted against the Cts1 protein levels. *B,* average CTP levels are plotted against the average Cts1 protein levels. *C,* average CTP levels are plotted against the average *Cts1* mRNA levels. The sphere volume in each strain corresponds to the average values of Cts1 protein levels (*A* and *B*), and to the average values of *Cts1* mRNA levels (*C*), as calculated after three biological repeats. *Cts1* mRNA levels, Cts1 protein levels, and CTP levels were calculated as described in Figs. 6, 5, and 8, respectively. All TOR mutant strains were constructed in a Cts1–YFP background.

We subsequently examined whether the effect of Gad8 S6/AGC kinase, Crf1 transcriptional corepressor, and Tor1 kinase on the regulation of cytoophidia formation via altered protein abundance originates at the transcriptional level. To test this, we extracted RNA from *gad8*Δ, *crf1*Δ, and *tor1*Δ knockout mutant strains, as well as from several others, and we measured the *Cts1* transcript levels by qPCR. Our analysis showed that the *Cts1* transcript levels are not significantly changed among any of the TORC1 or TORC2 mutants (*p* > 0.5), compared with the control strain (Fig. 6*A*). Furthermore, the *Cts1* mRNA content did not correlate with the changes in the average length of cytoophidia (Fig. 6*B*) nor did it with the Cts1 protein levels (Fig. 7*A*).

### Absence of Gad8 or Crf1 leads to reduced Cts1 enzymatic activity

Cts1 catalyzes the rate-limiting step in the production of CTP (3, 4). The main function of CTPS is to catalyze the last step in pyrimidine nucleotide biosynthesis. Filament formation potentially regulates CTPS activity and therefore CTP homeostasis. It has been shown that Cts1 enzymatic activity is correlated to CTP synthase's ability to form filaments, but the effect can differ depending on the system. In *Escherichia coli,* large-scale filament formation inhibits Cts1 activity (22, 29), similar to what has been observed in *S. cerevisiae* (23). However, Cts1 filamentation promotes its catalytic activity in human cells (29).

**Figure 8.**
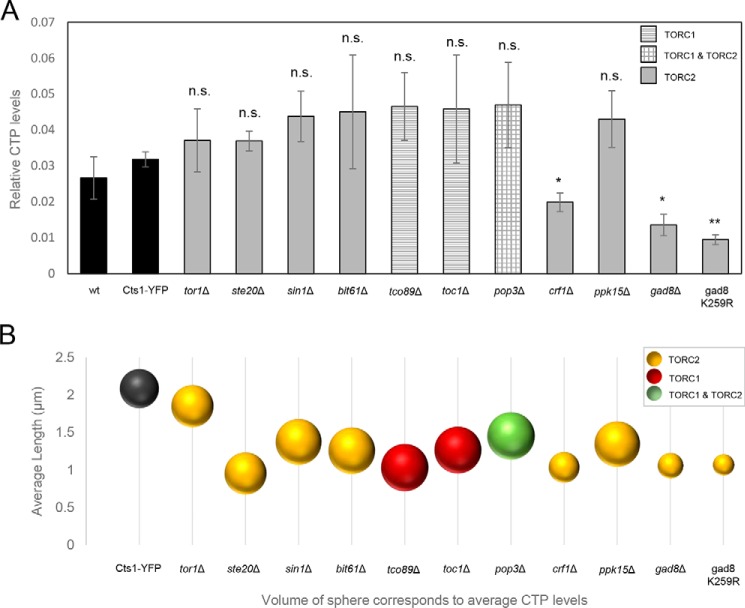
**Shorter cytoophidia phenotype in TOR mutants is not dependent on Cts1 enzymatic activity.** *A,* exponentially growing cells in rich medium were collected, washed with glucose, and treated with TCA. CTP levels were measured by UPLC. The relative quantity of CTP in each strain was plotted. All TOR mutant strains were constructed in a Cts1–YFP background. The experiment was performed in triplicate. *Error bars* show the mean ± S.D.: as calculated from three independent repeats (**, *p* < 0.01; *, *p* < 0.05; *n.s.*, not significant). The *different column patterns* indicate participation in TORC1, TORC2, or both complexes, as noted. *B,* the average length of cytoophidia was calculated as described in Figs. 2*C* and 3*C* for all TOR mutants. It was then plotted against the average CTP levels contained in an equal number of cells for each mutant, as measured by UPLC. The sphere volume in each case corresponds to the average values of CTP levels, as calculated after three biological repeats. All TOR mutant strains were constructed in a Cts1–YFP background.

Thus, we wanted to investigate whether the reduction in the average length of CTPS filaments in TOR mutant strains is correlated with a change in Cts1 enzymatic activity. To address this question, we used ultra-performance LC (UPLC) to measure the relative CTP levels in TOR knockout mutant strains, before plotting them against the CTP levels in the control WT strain (Cts1–YFP) (Fig. 8*A*). The CTP pool homeostasis was significantly disrupted in particular mutants (*crf1*Δ, *gad8*Δ, and gad8 K259R) but did not change significantly in others (*tor1*Δ, *ste20*Δ, *sin1*Δ, *bit61*Δ, *tco89*Δ, *toc1*Δ, *pop3*Δ, and *ppk15*Δ) (Fig. 8*A*). Notably, in the TOR downstream effector mutants *crf1*Δ, *gad8*Δ, and gad8 K259R, the Cts1 enzymatic activity was reduced compared with the WT (Cts1–YFP) strain (relative average value of 0.0318; S.D.: ±0.002) by 37.7% (*p* < 0.01) (0.0198; S.D.: ±0.0025), 57.5% (*p* < 0.01) (0.0135; S.D.: ±0.0029), and 70.3% (*p* < 0.01) (0.0094; S.D.: ±0.0013), respectively, which is in agreement with previous studies showing that TOR signaling promotes pyrimidine synthesis through S6K-mediated phosphorylation (71). However, the reduction in CTPS activity in *crf1*Δ, *gad8*Δ, and gad8 K259R strains does not seem to be specifically correlated with the reduction of the average length of cytoophidia, as several TOR knockout strains, such as *ste20*Δ and *tco89*Δ, show similarity to the *crf1*Δ, *gad8*Δ, and gad8 K259R mutants' reduction in the average length of cytoophidia, but not a significant reduction in the CTPS activity (Fig. 8*B*). Notably, the reduction in the CTP levels was correlated with the reduction of Cts1 protein levels for the *crf1*Δ, *gad8*Δ, and gad8 K259R strains (Fig. 7*B*) and was not correlated with the Cts1 mRNA levels in all TOR mutants tested (Fig. 7*C*). Overall, the reduction of Cts1 activity observed in knockout mutants of Gad8 TORC2 effector kinase and of the Crf1 transcriptional corepressor is plausibly the result of reduced protein levels; however, this does not seem to be the cause of the reduction observed in the average length of the CTPS filament.

## Discussion

Here, we established a link between Cts1 cytoophidia formation in *S. pombe* and its regulation by the TOR-signaling pathway. This demonstrated the conserved nature of such regulation from higher to lower eukaryotes, in agreement with our previous observations in mammalian and *Drosophila* cells (51). In fission yeast, we utilized a different approach by which we evaluated the effect that each TORC1 and TORC2 subunit has on cytoophidia formation, as well as the effects that their downstream effectors have on the regulation of Cts1 compartmentation. Single knockout mutants of most TOR subunits and downstream effector proteins resulted in reduced CTPS filamentation in *S. pombe* cells. In contrast to mammalian cells, in which only TORC1 is implicated in the regulation of Cts1 filamentation, in fission yeast both TORC1 and TORC2 complexes are involved. We showed that the regulation is mediated by S6K/AGC kinases that act downstream of both TORC1 and TORC2 complexes, contrary to mammalian cells, in which only mTOR1/S6K1 has been shown to play a role (51). In *S. pombe*, we further identified the Crf1 transcriptional corepressor, downstream of TORC2 subcomplex, as a major regulator of Cts1 filamentation. TOR signaling seems to be employing similar molecular and cellular pathways to regulate cell growth and Cts1 filamentation.

**Figure 9.**
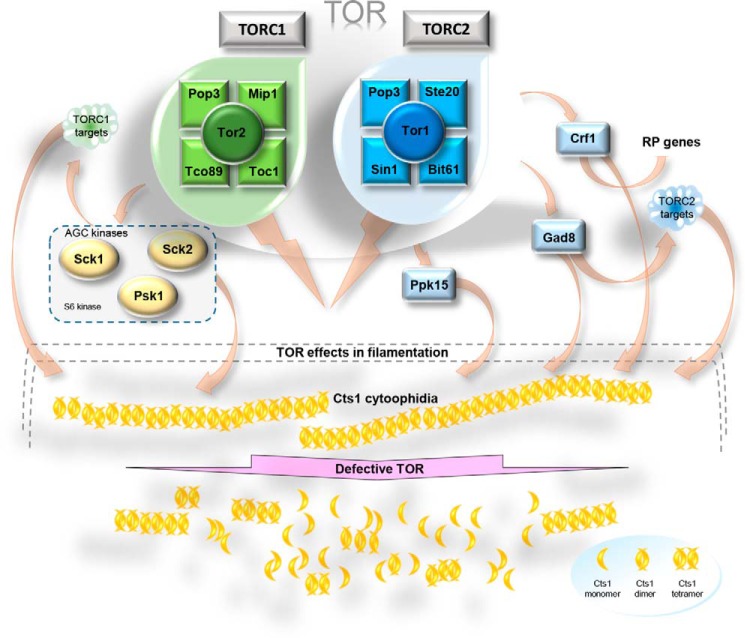
**Schematic model of cytoophidia regulation by TOR.** TOR complex in fission yeast consists of TORC1 and TORC2 sub-complexes, shown in *green* and *blue*, respectively. Under physiological conditions, exponentially growing cells exhibit normal Cts1 cytoophidia formation (depicted as *yellow* filaments). Proper cytoophidia formation is mediated by both TORC1 and TORC2 sub-complexes. Downstream effector proteins, such as the S6 kinases Gad8 (also an AGC kinase), Sck1, Sck2, and Psk1 mediate the proper formation of Cts1 cytoophidia, either by their immediate kinase action on CTPS or by their regulatory action on TORC1 and TORC2 targets. Crf1 transcriptional corepressor of ribosomal proteins also seems to play a key role in Cts1 filamentation via the TORC2 pathway. When TOR signaling is inhibited by drugs or does not function properly, such as in knockout mutants of TOR subunits or effector proteins, the Cts1 cytoophidia become significantly shorter and/or dissociate (*bottom*). *RP,* ribosomal protein; *AGC,* family of kinases named after three of its members: PKA, PKG, and PKC.

Our data indicate that the regulation of cytoophidia formation is implemented predominantly by effector kinases acting downstream of both TORC2 and TORC1 pathways, namely by Gad8 S6/AGC kinase and Ppk15 Ser/Thr kinase, as well as Sck1, Sck2, and Psk1 S6 kinases. As demonstrated, in the absence of Gad8 effector kinase, we observed a significant ∼50% reduction in the number of cells containing cytoophidia, along with an ∼50% reduction in the average length of Cts1 filaments, whereas the respective reduction in *ppk15*Δ was ∼36% (Figs. 4*D* and 3*C*). Similarly, TORC1 pathway's Sck1, Sck2, and Psk1 S6 kinases showed an ∼33–56% reduction in the average length of cytoophidia (Fig. 3*C*). Previous studies in *S. cerevisiae* and mammalian cells have shown that CTPS can be regulated via phosphorylation by kinases (72–76). The C-terminal region of CTPS in mouse fibroblasts has been shown to have numerous phosphorylation sites (71), and we have previously demonstrated that a phospho-specific antibody against *Drosophila* CTPS Ser-36 can recognize Cts1 filaments (15). Furthermore, when the conserved, post-translationally modified (included by phosphorylation) CTPS N-terminal domain in a *Drosophila* mutant was deleted, the cytoophidium assembly was affected (77). Knockdown of the mTORC1 downstream target S6K1 can inhibit CTPS filamentation, whereas S6K1 overexpression can reverse the disassembly of cytoophidia caused by the mTOR knockdown (51). In agreement with these, our results suggest a plausible regulation of CTPS compartmentation by TOR kinase pathway in *S. pombe* (Fig. 9). Future studies to determine the detailed mechanism of Cts1 phosphorylation should help elucidate the direct or indirect mode of cytoophidia regulation by TOR, as according to our data on fission yeast, as well as data on mammalian cells (51), the nature of the regulation seems to be very dynamic.

Importantly, we have demonstrated that deletion of Crf1 transcriptional co-repressor shows 95.3% reduction in cells containing cytoophidia and ∼50% reduction in their average length (Figs. 4*D* and 3*C*). Crf1 is a transcriptional corepressor for ribosomal proteins via TOR signaling and is orthologous to *S. cerevisiae* Crf1, also involved in the repression of ribosomal protein gene transcription by TOR (59, 60). The strong effect of *crf1*Δ on CTPS filamentation presents another dimension in the regulation of cytoophidia formation, involving the regulation of ribosomal proteins at the transcription level and the protein production machinery of the cell. Because the knockout mutant of *Crf1* causes an almost complete abolishment of Cts1 filamentation, it is worth exploring this mode of regulation further.

Even though CTPS protein expression does not change significantly in most of the TOR mutants checked, it does reduce considerably in the *tor1*Δ mutant, as well as in *gad8*Δ, gad8 K259R, and *crf1*Δ strains (Fig. 5), suggesting that, especially for the TORC2 downstream effector knockout mutants, the regulation of Cts1 filamentation could be mediated via the reduction of Cts1 protein levels. Notably, the regulation of CTPS filamentation by TOR does not seem to be mediated via reduced expression of CTPS protein in mammalian cells (51), suggesting a differential mode of regulation between higher and lower eukaryotes, at least for the TORC2 downstream effector proteins. Furthermore, the *gad8*Δ, gad8 K259R, and *crf1*Δ strains showed reduced Cts1 activity (Fig. 8*A*), which could be attributed to the lower protein levels. However, the shorter cytoophidia phenotype does not seem to be correlated to the Cts1 enzymatic activity *per se* (Fig. 8*B*). It is known that *de novo* pyrimidine synthesis is specifically stimulated by growth signaling through mTOR and S6K1 and not by the salvage pathway (71, 78). Thus, we argue that in our TOR mutant strains in *S. pombe*, the pyrimidine synthesis is similarly regulated via the *de novo* pathway through CTP synthase. It would nevertheless be interesting to examine whether the salvage pathway may contribute to the changes in CTP levels observed in Gad8 and Crf1 mutants by using different isotopes of carbon (22, 79–81). Interestingly, polymerization of CTPS in cytoophidia inhibits the enzyme's catalytic activity in *S. cerevisiae* and *E. coli* (22, 23, 29), while it promotes it in mammalian cells (29). In *S. pombe*, reduction of filamentation did not significantly change the CTPS enzymatic activity in the TOR subunit knockout mutants, while it reduced it in the TORC2 downstream effector mutants. This suggests that in the case of Crf1 and Gad8 mutants, CTPS polymerization increases the enzyme's activity. Alternatively, this could be the result of indirect effects caused by the dysfunctional TOR pathway. It would be interesting to elucidate the specifics of how Cts1 filamentation is correlated with the activity of CTPS in fission yeast.

It should be stressed that cytoophidia formation in the multiple TOR mutants is not correlated to a possible stall of the cells in a particular phase of the cell cycle in the growth conditions used in our experiments. Our cell cycle analyses performed on a series of log-phase cultures of TOR mutants showed no particular over-representation of cells in a specific cell cycle phase compared with the control strain (Fig. S2), whereas our manual measurement of cytoophidia length and the detection of the presence or absence of filaments was unbiased by the cell cycle phase. Additionally, the growth rate of TOR mutants did not change significantly in physiological conditions, with the exception of Gad8 mutants (*p* < 0.05) (Fig. S3). We ensured a no-bias by changes in the growth rate by conducting our measurements and observations in cells collected from cultures of the same log-phase.

Recently, there has been an increase in the number of proteins reported to form cytoplasmic structures, suggesting that filament formation is another level of control for highly-regulated proteins (17, 20, 82, 83). The importance of Cts1 filamentation, in particular, has been highlighted by a number of studies over the last few years (16, 21, 23–26, 32), and possible functional roles have been proposed (28). It has been hypothesized that the presence of this feature across distantly related species should represent a conserved regulatory strategy in the production pathway of CTP (27). However, the mode of Cts1 filament regulation has remained relatively elusive, until we recently showed that cytoophidium assembly in mammalian cells is mediated by the mTOR–S6K1 pathway (51). In this study, we demonstrated that this mode of regulation is conserved in the lower eukaryotic organism *S. pombe*, but, in contrast to higher eukaryotic systems, the effect on CTPS filamentation is mediated by both TORC1 and TORC2 pathways, instead of just by TORC1, indicative of an earlier stage of regulation during evolution. According to our findings, the CTPS cytoophidium assembly dynamics are significantly affected by TORC1 and TORC2 downstream effector proteins, such as Gad8, Sck1, Sck2, and Psk1 kinases, and we further identified Crf1 transcriptional corepressor of ribosomal proteins as a major regulator of Cts1 filamentation. Considering that dysregulated CTPS levels and increased metabolic activity are characteristics of multiple forms of cancer (5, 10–12, 41, 84, 85), and that both CTPS and TOR are often used as targets in anti-cancer drug development, a better understanding of how cells utilize and regulate CTPS becomes imperative. To this direction, further exploration of the particulars of TOR's effect on Cts1 cytoophidia formation could prove crucial.

## Experimental procedures

### Yeast strains and culture conditions

All viable deletion strains of TORC1, TORC2, and selected downstream mediator proteins were constructed and are summarized in Table 1. *S. pombe* cells were cultured in standard rich media (YE4S; YES enriched with supplements leucine, uracil, histidine, and adenine at 100 μm). Drug inhibition of the TOR pathway was achieved with the use of rapamycin and everolimus TOR inhibitors at a concentration of 1 μm for 4 h in exponentially growing cultures. To construct the Cts1–YFP strain (JLL005S), JLL003S WT strain was transformed with plasmid pSMUY2-Ura4-cts1-YFP after linearization with SpeI, as described previously (19). More specifically, genomic DNA was used as a template to amplify the *Cts1* gene by PCR, using primers containing ApaI and XhoI restriction sites. The *Cts1* fragment and pSMUY2+ plasmid were then digested using ApaI and XhoI restriction enzymes (New England Biolabs), and the *Cts1* fragment was subsequently ligated into the plasmid using T4 DNA ligase (New England Biolabs). The resulting plasmid was amplified, linearized with SpeI, and transformed into the JLL003S strain, using the lithium acetate method (Paul Nurse Lab manual). The resulting Cts1–YFP strain (JLL005S) expresses Cts1–YFP under the control of the endogenous promoter at the endogenous locus. The Cts1–YFP strain has the same growth profile as the WT strain (19). Haploid TOR deletion strains containing Cts1–YFP were obtained by PCR-based gene targeting using pFA6A-KanMX6 plasmid as template for specially-designed gene-specific 80-bp-long primers, as described previously (86), followed by transformation of Cts1–YFP strains. Briefly, in the PCR-based gene-targeting method used to obtain the deletion strains, forward and reverse primers were designed to be complementary to a 20-bp region in each side of the *kan*^r^ cassette included in the pFA6A-KanMX6 plasmid. Additional flanking 60 bp sequences, complementary to the immediate 5′-upstream and 3′-downstream endogenous region of the gene of interest, were included in each primer (total length of 80 bp for each primer). The primers were used to a PCR with the pFA6A-KanMX6 plasmid as template to amplify the *kan*^r^ cassette. The PCR product contained the *kan*^r^ cassette flanked by 60 bp on each of the 5′- and 3′-ends, complementary to the endogenous region of the gene of interest. The PCR product was then purified and ∼1–2 μg were transformed into the Cts1–YFP strain (JLL005S), using the lithium acetate method (Paul Nurse Lab manual). Single colonies obtained by the transformation were then screened by PCR to confirm both the absence of each gene and the replacement of the gene by the selection cassette in the targeted genetic locus. The Cts1–YFP tagging in the *gad8::ura4*+ and gad8 K259R mutants was done by transforming strains JLL149 and JLL150 with plasmid pSMUY2-Ura4-cts1-YFP after linearization with SpeI, as described above. For the construction of deletion strains where the selection was hygromycin B or nourseothiricin, the plasmids pFA6a-hphMX6 and pFa6a-natMX6 were used in PCR-based gene targeting, respectively. These deletion strains were similarly verified by PCR as described above. Table S1 contains a list of the primers used in the construction and verification of the strains. *gad8*Δ and K259 strains (without Cts1–YFP) were a gift by Janni Petersen.

**Table 1:** ***S. pombe* strains**

Strain no.	Genotype
JLL003S	h− *his3*-D1 *ura4*-D18 *leu1*-32 *ade6*-M216
JLL005S	h + cts1-YFP (ura4 + ) *ura4*-D18 *leu1*-32 *ade6*-M216
JLL056	h + cts1-YFP (ura4 + ) *toc1*::KanMX6 *ura4*-D18 *leu1*-32 *ade6*-M216
JLL091	h + cts1-YFP (ura4 + ) *tco89*::KanMX6 *ura4*-D18 *leu1*-32 *ade6*-M216
JLL081	h + cts1-YFP (ura4 + ) *pop3*::KanMX6 *ura4*-D18 *leu1*-32 *ade6*-M216
JLL092	h + cts1-YFP (ura4 + ) *sin1*::KanMX6 *ura4*-D18 *leu1*-32 *ade6*-M216
JLL057	h + cts1-YFP (ura4 + ) *ste20*::KanMX6 *ura4*-D18 *leu1*-32 *ade6*-M216
JLL067	h + cts1-YFP (ura4 + ) *tor1*::KanMX6 *ura4*-D18 *leu1*-32 *ade6*-M216
JLL119	h + cts1-YFP (ura4 + ) *ppk15*::KanMX6 *ura4*-D18 *leu1*-32 *ade6*-M216
JLL054	h + cts1-YFP (ura4 + ) *crf1*::KanMX6 *ura4*-D18 *leu1*-32 *ade6*-M216
JLL073S	h + cts1-YFP (ura4 + ) *bit61*::KanMX6 *ura4*-D18 *leu1*-32 *ade6*-M216
JLL149	h + cts1-YFP (KanMX6) *gad8*::ura4 + *ura4*-D18
JLL150	h + cts1-YFP (KanMX6) *gad8 K259R* (ura4 + ) *ura4*-D18
JLL093S	h + cts1-YFP (ura4 + ) *sck1*::kanMX6 *ura4*-D18 *leu1*-32 *ade6*-M216
JLL095S	h + cts1-YFP (ura4 + ) *sck2*::hygB *ura4*-D18 *leu1*-32 *ade6*-M216
JLL100S	h + cts1-YFP (ura4 + ) *sck1*::kanMX6 *sck2*::hygB *ura4*-D18 *leu1*-32 *ade6*-M216
JLL099S	h + cts1-YFP (ura4 + ) *psk1*::natMX6 *ura4*-D18 *leu1*-32 *ade6*-M216
JLL101S	h + cts1-YFP (ura4 + ) *sck1*::kanMX6 *sck2*::hygB *psk1*::natMX6 *ura4*-D18 *leu1*-32 *ade6*-M216

### Growth assays

Cells were grown in rich medium (YE4S) until reaching OD_600_ ∼1. Seven serial dilutions (1:5, unless mentioned otherwise) were spotted on plain YE4S agar plates or on YE4S plates containing rapamycin (S1039, Selleck Chemicals, China) or everolimus (S1120 Selleck Chemicals, China) TORC1 inhibitors at a final concentration of 1 μm and incubated at 30 °C for at least 2 days. Photos of the plates were taken at 24, 36, and 48 h, using a Nikon D3100 digital camera under white light.

For the construction of growth curves, exponentially growing cells were cultured in YE4S, or in YE4S containing rapamycin or everolimus TORC1 inhibitors at a final concentration of 1 μm, starting from an OD_600_ = 0.15. Changes in the optical density of the cultures were monitored every 1.5 h for a total period of ∼10 h. The experiment was repeated in triplicate before construction of the growth curves. Standard deviation was calculated after three biological repeats.

### Sample preparation, microscopy, and statistical analysis

Cts1 filaments in *S. pombe* are formed in exponentially growing cells. Cells in logarithmic phase were fixed by using 16% paraformaldehyde (PFA) solution for 10 min while still in liquid culture (final PFA concentration of 4%), followed by two washes with PBS buffer, before being mixed with YE4S mountant (1.2% LMT agarose in YE4S, 0.1 mm *N*-propyl gallate, to reduce phototoxicity, containing DAPI (Sigma, D9542) 200 ng/ml as DNA dye). Cells were visualized using a Zeiss Axio Imager 2 microscope, photographed with Zen 2 lite (blue edition) software, and processed with ZEN 2 lite and ImageJ software. For cell quantification, at least 400 cells were scored manually in each of three independent experiments. Two types of Cts1 cytoophidia are present in fission yeast, one longer in the cytoplasm and one shorter in the nucleus (19). No difference was observed between the cytoplasmic and nuclear filaments of Cts1 in the TOR mutants; either both were present in the individual cells scored or both were absent. The standard deviation was calculated from three biological replicates. The percentage value of cells containing cytoophidia in each mutant was calculated in each experiment, and a mean value from the three trials was obtained. A two-tailed homoscedastic *t* test was performed to evaluate the statistical significance of the mean value in each strain *versus* the mean value in the Cts1–YFP control strain. For the measurement of cytoophidia length, the length of at least 400 cytoophidia in each of three independent experiments was scored manually with the use of ImageJ software. Only the length of cytoplasmic cytoophidia was measured. The standard deviation was calculated from three biological replicates. A two-tailed homoscedastic *t* test was performed to evaluate the statistical significance of the mean value of cytoophidia length in the three replicates for each mutant strain *versus* the respective mean value calculated for Cts1–YFP strain.

For the immunostaining, cells were grown until mid-log phase, fixed by incubating with PFA at a final concentration of 4% for 10 min, while still in liquid culture (as described above), and washed twice with PBS buffer. Cells were then incubated with 0.2 mg/ml lyticase from *Arthrobacter luteus* (Sigma, L2524) in PBS for 20 min at 37 °C, in order to make the cell walls permeable, and washed twice with PBS. Subsequently, the cells were treated with 1% Triton in PBS for 30 s to render the cell membranes permeable and washed twice with PBS. Afterward, ∼5 × 10^6^ cells were applied on top of Titan positively-charged adhesion microscope slides to facilitate their sticking on the glass surface before incubation for 2 min at 40 °C on a heat block for fixation onto the slide surface. The slides were then put in a humid chamber, and cells were incubated by blocking solution (3% BSA in PBS) for 1 h. Incubation of cells on the slides with primary antibody was done overnight at 1:500 dilution in PBS, 3% BSA solution, using rabbit polyclonal IgG anti-CTP synthetase ½ (y-88) antibody with yeast specificity (sc-134457, Santa Cruz Biotechnology). The cells were then washed three times in PBS, before incubation with the secondary antibody at 1:500 dilution in PBS, 3% BSA solution for 1 h. The secondary antibody used was anti-rabbit Alexa 488 (A11008, Molecular Probes). The cells were subsequently washed three times with PBS, air-dried, and covered with YE4S mountant containing DAPI, before proceeding to fluorescent microscopy, as described above.

### Western blotting analyses

Protein extraction from an equal number of cells using the trichloroacetic acid (TCA) method and Western blotting were performed as described previously (87). Briefly, for the protein extraction, 4 × 10^6^ cells of exponentially growing cultures were collected and washed once with 400 μl of 1.2 m sorbitol. Subsequently, 100 μl of 20% TCA was added to the pellet along with micro glass beads (∼200 μl in volume) and protease inhibitor mixture (Sigma, P8215). The cells were then beaten with the glass beads for 8.5 min at 4 °C on a Vortex machine (30 s of beating followed by 30 s of resting time). 900 μl of 5% TCA was then added to the mix, before mixing briefly and collecting 800 μl of the supernatant to a new tube. The cell extract was then centrifuged for 15 min at 4 °C, and the supernatant was discarded. 50 μl of 1× Laemmli buffer was added to the cell pellet, which was then resuspended, before being boiled for 5 min and briefly centrifuged. 25 μl of the supernatant was loaded onto an 8% SDS-polyacrylamide gel and electrophoresed at 100 V. The semi-dry transfer of the proteins onto polyvinylidene difluoride membrane was done using the Trans-blot turbo system (Bio-Rad). For the detection of the YFP-tagged proteins, a goat polyclonal anti-GFP antibody (Abcam ab6673) was used. Tubulin was used as a loading control, and the probing was done with mouse monoclonal anti-α-tubulin antibody (Sigma, T5168). The secondary anti-goat antibody used was peroxidase-AffiniPure Donkey anti-goat IgG (Jackson ImmunoResearch, 705-035-147), and the secondary anti-mouse antibody used was anti-mouse IgG HRP-linked (Cell Signaling Technology, 7076). Detection of the protein signal was done by using SuperSignal West Pico Chemiluminescence Substrate (Thermo Fisher Scientific, 34087) and exposing the membrane in an Amersham Biosciences Imager 600 detector. Quantification of the protein bands' intensity was done with the use of ImageJ software. Standard deviation was calculated from three biological repeats. A two-tailed homoscedastic *t* test was performed to evaluate the statistical significance of the mean value of Cts1–YFP protein in the three replicates for each mutant strain *versus* the respective mean value calculated for the control Cts1–YFP strain.

### Real-time and RT-PCR

RNA was extracted using TransZol Up reagent (Tran ER501-01-01). Briefly, 5 × 10^7^ log-phase cells were collected by centrifugation and resuspended in 400 μl of TransZol Up reagent. After a 5-min incubation, the solution was extracted with 80 μl of phenol/chloroform. The RNA was precipitated after adding 200 μl of isopropyl alcohol and centrifuging at 4 °C for 20 min at 13000 rpm. The pellet was washed with 75% ethanol, air-dried for 5–10 min, resuspended in 35 μl of RNase-free water, and heated at 60 °C for 15 min. RT was performed to 1 μg of RNA per sample using PrimeScript^TM^ RT Mastermix (Takara RR036A), according to the product specifications, and transcript enrichment was calculated by qPCR performed in QuantStudio^TM^ 7 Flex (Applied Biosystems), using SYBR Green (Bimake B21702) to monitor the reaction. Table S1 contains the sequences of the primers used in the qPCR. Normalization of the expression levels was done over a constitutively-expressed gene (*Act1*). A two-tailed homoscedastic *t* test was performed to evaluate the statistical significance of the mean value of *Cts1* mRNA level in the three replicates for each mutant strain *versus* the respective mean value calculated for the control Cts1–YFP strain, after normalizing over the *Act1* mRNA levels.

### Measurement of CTP pools

Preparation of samples for CTP level measurements was done as described previously (88) with some modifications in the protocol. Briefly, strains were grown to log phase in YE4S, and 5 × 10^8^ cells were collected by centrifugation and washed once with 3% glucose solution. The cell pellet was kept on ice, and 50 μl of 10% TCA solution was added with brief stirring. Samples were immediately frozen at −80 °C before proceeding to UPLC analysis. Thawed samples were centrifuged, and 20 μl of the supernatant was mixed with 80 μl of milli Q water (final concentration of 2% TCA). Nucleotides were neutralized by extracting the samples with 100 μl of FREON (1,1,2-trichlorotrifluoroethane)/trioctylamine (4:1). After centrifugation, the supernatant (∼100 μl) was used for the UPLC.

Chromatographic analysis was performed using an Ultimate 3000 UPLC system (Dionex) with diode array detection and monitoring at 254 nm. Precipitated and extracted samples were injected into an ACQUITY UPLC HSS T3 column (100 × 2.1 mm, 1.8 μm; Waters) at 35 °C. The mobile phase A consisted of 10 mm potassium dihydrogen phosphate, 10 mm tetrabutylammonium hydroxide, and 10% methanol at pH 6.9, and mobile phase B consisted of 50 mm potassium dihydrogen phosphate, 6 mm tetrabutylammonium hydroxide, and 30% methanol at pH 7. The injection volume was 5 μl. The flow rate was 0.15 ml/min, and elution conditions at 35 °C were applied with a linear gradient as follows: 0–5 min, 25–45% B; 5–40 min, 45–85% B; 40.1–45 min, 25% B. This chromatographic approach allowed an effective separation of the different NTPs, which was followed by relative quantification.

### Flow cytometry

10^7^ cells of each exponentially growing culture were collected by centrifugation, and the pellet was resuspended in 1 ml of 70% ethanol. 0.3 ml of the cell suspension was added to ∼4.5 ml of 10 mm EDTA, pH 8.0, mixed, and spun down. The cell pellet was then resuspended to 0.5 ml of 10 mm EDTA, pH 8.0, containing 0.1 mg/ml RNase A (Axygen), and incubated for 2 h at 37 °C. The cells were then stained with Sytox Green fluorescent dye (Invitrogen, S7020) at a final concentration of 1 μm. Subsequently, the cells were briefly sonicated (45 s, power 25 watts, Scientz-IID Ultrasonic Homogenizer), before flow cytometry analysis was conducted using a flow cytometer (BD LSRFortessa). 30,000 cells per sample were measured in each trial. Construction of DNA-W/DNA-A cytograms was done after analysis with FlowJo 7.6.1 software. As calculated for fission yeast, and because of the rod-like shape of fission yeast cells, a cell containing two nuclei, which sequentially pass through the excitation focus, emits a DNA fluorescent signal that lasts longer than a mononuclear cell with a narrow field of excitation (89). This is because the DNA labeled by Sytox Green will remain for a longer period of time in the excitation focus for cells with two nuclei *versus* cells with one nucleus. Thus, the DNA signal (DNA-W) is expected to last for a longer period of time in a binuclear *versus* a uninuclear cell (89). In contrast, the total area of the DNA signal (DNA-A), representing the total DNA content of the cell passing the excitation focus, should remain unaffected by nuclear distribution (89). Based on this, using DNA-W/DNA-A cytograms, we were able to visualize four different subpopulations of cells and calculate the average percentages of cells in the different phases of the cell cycle for the TOR mutants and for the control strain, as described in Fig. S2. The standard deviation was calculated after two biological repeats.

## Author contributions

C. A., L. H., and J.-L. L. conceptualization; C. A. and L. H. formal analysis; C. A., L. H., K. W., and J.-L. L. investigation; C. A. and L. H. methodology; C. A. writing-original draft; C. A. and J.-L. L. writing-review and editing; J.-L. L. resources; J.-L. L. supervision; J.-L. L. funding acquisition; J.-L. L. project administration.

## Supplementary Material

Supporting Information

## Footnotes

2 The abbreviations used are:

CTPS: CTP synthase
TOR: target of rapamycin
mTOR: mammalian target of rapamycin
qPCR: quantitative PCR
L: length
UPLC: ultra-performance liquid chromatography
PFA: paraformaldehyde
DAPI: 4′,6-diamidino-2-phenylindole
S6K: S6 kinase.
