# Supplementary material for: The TOR pathway modulates cytoophidium formation in Schizosaccharomyces pombe
Source: J Biol Chem. 2019 Aug 19;294(40):14686–703. doi: 10.1074/jbc.RA119.009913 (PMC6779450; doi:10.1074/jbc.RA119.009913)
Supplement: Supporting Information [file supp_294_40_14686__index.html]

The TOR pathway modulates cytoophidium formation in Schizosaccharomyces pombe — TOR pathway affects Cts1 filamentation — Supporting Information 

# The TOR pathway modulates cytoophidium formation in *Schizosaccharomyces pombe*

## Supporting Information

- Supporting Information (to be published online) - Supplementary Table S1 and Supplementary Figures S1-S3
