# Supplementary material for: The TOR pathway modulates cytoophidium formation in Schizosaccharomyces pombe
Source: J Biol Chem. 2019 Aug 19;294(40):14686–703. doi: 10.1074/jbc.RA119.009913 (PMC6779450; doi:10.1074/jbc.RA119.009913)
Supplement: Supporting Information [file supp_RA119.009913_153693_2_supp_376574_pw0209.pdf]

The TOR pathway modulates cytoophidium formation in *Schizosaccharomyces pombe*

ChristosAndreadis[1], LydiaHulme[2], KatherineWensley[2], and Ji-LongLiu[1,2]*

[1]School of Life Sciences and Technology, ShanghaiTech University, 201210, Shanghai, China
[2]MRC Functional Genomics Unit, Department of Physiology, Anatomy and Genetics, University of Oxford, Oxford, OX1 3PT, UnitedKingdom

Running Title: *TOR pathway affects Cts1 filamentation*

*To whom correspondence should be addressed: Ji-Long Liu: School of Life Sciences and Technology, ShanghaiTech University, 201210, Shanghai, China; Department of Physiology, Anatomy and Genetics, University of Oxford, Oxford, OX1 3PT, United Kingdom; liujl3@shanghaitech.edu.cn; jilong.liu@dpag.ox.ac.uk; Tel.+86-21-20684533.

Supporting Information

Supplementary Table S1

Supplementary Figures S1-S3

Supplementary Table S1: List of primers

Primer Name	Sequence (5'-3')	Use
ppk15 delta F	GAGTGTGAATGAAGGCACTCGTATTCTGCCTATACTGATATATTCTTTGCAATACTTTACAAAAAGGCGTCTCTATTCATCGGATCCCCGGGTTAATTAA	construction of *ppk15*Δ strain
ppk15 delta R	TAAATTTGTAAATGCTTTATACTATTTACTATTGAAAACTGCATATTGAAGCCTCGAAGGAACTTTGAAAATATTCGTAGGAATTCGAGCTCGTTTAAAC	construction of *ppk15*Δ strain
ppk15 check F	AAGTGTTTCCCTTTGTTCCAGA	verification of *ppk15*Δ strain
ppk15 check R	TTGGCGATTTGTGAATGTCTAC	verification of *ppk15*Δ strain
crf1 delta F	GAACTAGAGGCTAAGAATTCCGCTTTCAAGCAGTTGAGTACTTAACCACAGCCAAGTAATTCGTCTTTGCGAATGTTAGGCGGATCCCCGGGTTAATTAA	construction of *crf1*Δ strain
crf1 delta R	AACTGGTGATAATAGGGAATCCTTGATTACATAGTTGCAACAAAGCAAGTTCAAAGACCAAATGAAAGCAAAGTGATCAAGAATTCGAGCTCGTTTAAAC	construction of *crf1*Δ strain
crf1 check F	AGGGTAGGGTAGTTGGTGGAAT	verification of *crf1*Δ strain
crf1 check R	ACAAAGAAAACCAAAGCCAAAA	verification of *crf1*Δ strain
sin1 delta F	GTATGGTCGCTAAGTATTCAAACTTCGTTGAAATTAAATATTTTATAAAGTCTGTAAATGCGATATAATAAGGACTCAGACGGATCCCCGGGTTAATTAA	construction of *sin1*Δ strain
sin1 delta R	CTGATGGTGATACGGAAATGCAAGAAAGTAAAAAGAATGTTATTATTAATGGATTTAATGAGGACGATGAAGACGATGAAGAATTCGAGCTCGTTTAAAC	construction of *sin1*Δ strain
sin1 check F	GTACTTTACGGTGTTACCCCCA	verification of *sin1*Δ strain
sin1 check R	ACTTCTTAGACGTTACGACGGC	verification of *sin1*Δ strain
ste20 delta F	CGCGTGTTGCACGCAATAAGTATTAGTACGAAAACATACTATCCCTTTATAAGGCTATTTCATGAAGGCGCTTATGGAAGCGGATCCCCGGGTTAATTAA	construction of *ste20*Δ strain
ste20 delta R	AGCGCAATCACCTTTTATGTAAACACTAGTCTAACTTAGTAAATAAACTTATGAAAAGAAACAAAGAGGGAAGGTCAAAGGAATTCGAGCTCGTTTAAAC	construction of *ste20*Δ strain
ste20 check F	CACTCCATGTCGTACAATCGTT	verification of *ste20*Δ strain
ste20 check R	TTACTATCGTATGGAGGCCAGG	verification of *ste20*Δ strain
tco89 delta F	CAAGCGTTTTGCTTGTATTCGTTTCTTCTTCATTTTCTTTCTAAACAGTTCCCCAAAGAACCCTCATTTAGACGTTTAGTCGGATCCCCGGGTTAATTAA	construction of *tco89*Δ strain
tco89 delta R	AGTATACAGATTACACATTTAGTTTGCTCCAAGGACTTCGGTAGATGGACATACAGGTATTTAGGACTTTAGCAATGACTGAATTCGAGCTCGTTTAAAC	construction of *tco89*Δ strain
tco89 check F	ATTTGCCATCTTTTGTTCGTTT	verification of *tco89*Δ strain
tco89 check R	GGGTAATCCCTCAACCCTTTAC	verification of *tco89*Δ strain

pop3 delta F	AGATCGCCAATCGTGCGTTTACAAGTCATGCTACAAAAAATTTACTAAATATATAATATAAACATATTTAATTGATTTTACGGATCCCCGGGTTAATTAA	construction of *pop3*Δ strain
pop3 delta R	TTTGTAAGTGACTAAGAAATCGTCTTGAAATCAGACACACTTAATATAAACGAATGTTGACAAGACAAGACTCTTGTTTTGAATTCGAGCTCGTTTAAAC	construction of *pop3*Δ strain
pop3 check F	ATTTGCCTTAACCTTTCGTCCT	verification of *pop3*Δ strain
pop3 check R	CTCCGAGATATGAAACTAGCAAA	verification of *pop3*Δ strain
toc1 delta F	CGGCGTACCTAATTCTTGCCAACGCCGATCTCACTGAGCAACGCGATTCGAGCGTTGACGAAATCTTCATAGTAGATTTACGGATCCCCGGGTTAATTAA	construction of *toc1*Δ strain
toc1 delta R	ATACTACCTTGTTTATGCAGGTAGTTCGATCGAATACTGTCGAGCATGTTTATAATCGGCTGTCGATCATACAAATCAAAGAATTCGAGCTCGTTTAAAC	construction of *toc1*Δ strain
toc1 check F	TGGACCTTGAATCCACCTTAGT	verification of *toc1*Δ strain
toc1 check R	TCGTCTGTTCTTAGGGTGTTGA	verification of *toc1*Δ strain
tor1 delta F	ATTGTGATGAATGCCTAAGTGGAAGAATTGAACACCGCGACTATTAGAAAGTCTATCGTTTCACTCGCTCTCTTTGATTCCGGATCCCCGGGTTAATTAA	construction of *tor1*Δ strain
tor1 delta R	AATAGTCATCCAGGAAAAGAATCATAACTTATTTGGAGCTCAGAAACGAGCGAATTTATAGACATAAATTAATAACAACAGAATTCGAGCTCGTTTAAAC	construction of *tor1*Δ strain
tor1 check F	ATTTAATCAAACTGGAAGGCCA	verification of *tor1*Δ strain
tor1 check R	ACGCTAAAGAAATTTGCTCCAA	verification of *tor1*Δ strain
bit61 delta F	CATTTTATAATGATTGAACAATGAATATCATCTCATGCTTATTTTGAAGAATCGCATTTCGCGTAACTAAAAGCAAATTTCGGATCCCCGGGTTAATTAA	construction of *bit61*Δ strain
bit61 delta R	TTGTGGACATAAACAAAAGCTGATCGTTAACTAATGATGAAAACAAAAGACTGGGAATATCAAGTATTGACACAAATTTTGAATTCGAGCTCGTTTAAAC	construction of *bit61*Δ strain
bit61 check F	ATTTAAGGCGCACATCGATACT	verification of *bit61*Δ strain
bit61 check R	GATCCGACTGGTACGATTGATT	verification of *bit61*Δ strain
sck1 delta F	ATCCGTTCGCTAAGATATCGTACTCCCGGATGTTACCACACTTTTCGATTAAGGGAATTGCGAAGGTTACATATGCGAAACGGATCCCCGGGTTAATTAA	construction of *sck1*Δ strains
sck1 delta R	AAACACGGAGCGAAGCCTTTGAGAGACAAAATAACTATAACGAAAGGGGAACATAATACATAATAAGTTATCTTCTATTGGAATTCGAGCTCGTTTAAAC	construction of *sck1*Δ strains
sck1 check F	CGCCCCTCTGATATTATAACCA	verification of *sck1*Δ strains
sck1 check R	AAGCAAAACACCCACAGTTTGC	verification of *sck1*Δ strains
sck2 delta F	ATATCCAACTGAGGTGAATTATTACTTGAGACTTTACATCATTAAATTTTATGCAGGTATTAAGTCGTGATTATATCAGTCGGATCCCCGGGTTAATTAA	construction of *sck2*Δ strains

sck2 delta R	TCGGGTTCAGGGATCCAGCCTAGAAAAAATATGGGCATAAGATTCTCAATGCAGTGATACATTCGTGGAGACCAAACGTAGAATTCGAGCTCGTTTAAAC	construction of *sck2*Δ strains
sck2 check F	ACTAGGGGTTGTTTCTTTGCAC	verification of *sck2*Δ strains
sck2 check R	TGTTGAAATTTTGCTGGACAAC	verification of *sck2*Δ strains
psk1 delta F	GTAGTAAGTTGATAACTTTAATTAGTAAAGAGGGCGAATCAAAAGCTTTATATATATCTAAACTTTACTAAATTGGCCAACGGATCCCCGGGTTAATTAA	construction of *psk1*Δ strains
psk1 delta R	GAAAACGATTAATTAATGGAGTTTCTTCAAGTATTATGAGTCCCTAAAGGATGAAAGGAGTTATGATATAGTGAGAATAAGAATTCGAGCTCGTTTAAAC	construction of *psk1*Δ strains
psk1 check F	TAGCCTATGTAAGCGGTCCAAT	verification of *psk1*Δ strains
psk1 check R	CACTAAGCTGACCGATGTTTTG	verification of *psk1*Δ strains
kanR	CGGATGTGATGTGAGAACTGTATCCTAGC	verification of strains constructed with geneticin selection
kanF	CGCTATACTGCTGTCGATTCG	verification of strains constructed with geneticin selection
natR	GTACGAGATGACCACGAAGC	verification of strains constructed with nourseothiricin selection
hygF	GTCTGGACCGATGGCTGTGTAG	verification of strains constructed with hygromycin B selection
hygR	CGTCAGGACATTGTTGGAGCCG	verification of strains constructed with hygromycin B selection
Cts1 ORF F	CAAGACTGGGTGGAGCGTGTTG	qPCR for *Cts1* transcription analyses
Cts1 ORF R	GCACCTGTTCGGGACCAACATG	qPCR for *Cts1* transcription analyses
Act1 ORF F	CACGGTATTGTCAACAACTGGGATG	qPCR for *Act1* transcription analyses
Act1 ORF R	GTAGTCAGTCAAGTCACGACCGG	qPCR for *Act1* transcription analyses

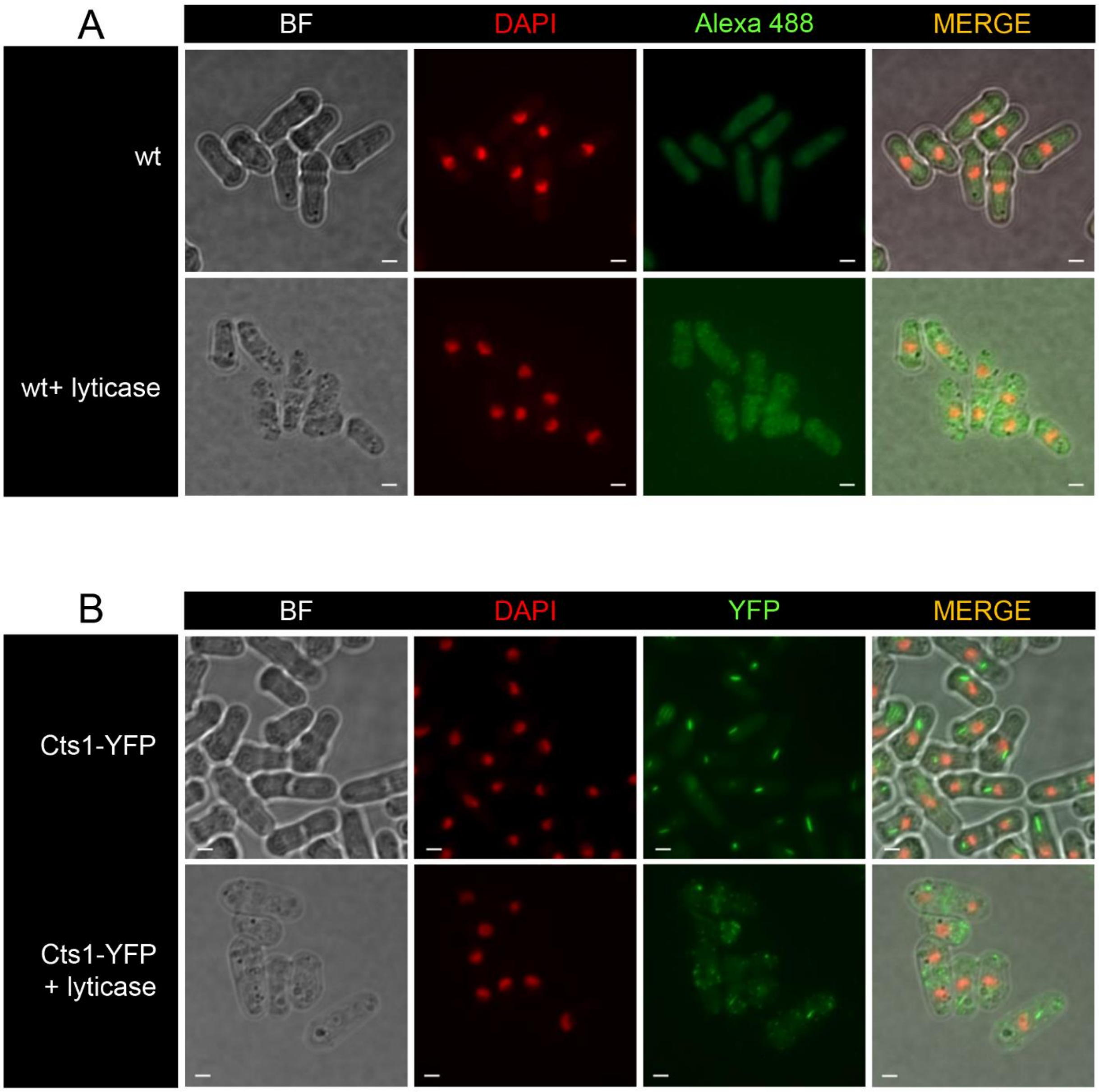

Figure S1. Immunostaining of wild type cells against CTP synthase is hindered by the effect of enzymatic breakage of cell walls on cytoophidia. (A) Wild type cells were grown in rich medium until exponential phase, followed by PFA fixation, immunostaining with anti-CTP synthetase ½ (y-88) antibody and Alexa 488 fluorescent dye, and fluorescence microscopy. The first row of panels show representative microscopic images of cells not treated with lyticase, with intact cell walls, restricting the entrance of the antibody. In the second row of panels, the cells were treated with lyticase, allowing the antibody to enter. (B) Representative microscopic images of Cts1-YFP cells grown in rich medium until exponential phase, fixed by PFA, and treated or not treated with lyticase, as indicated. Scale bar: 2μm.

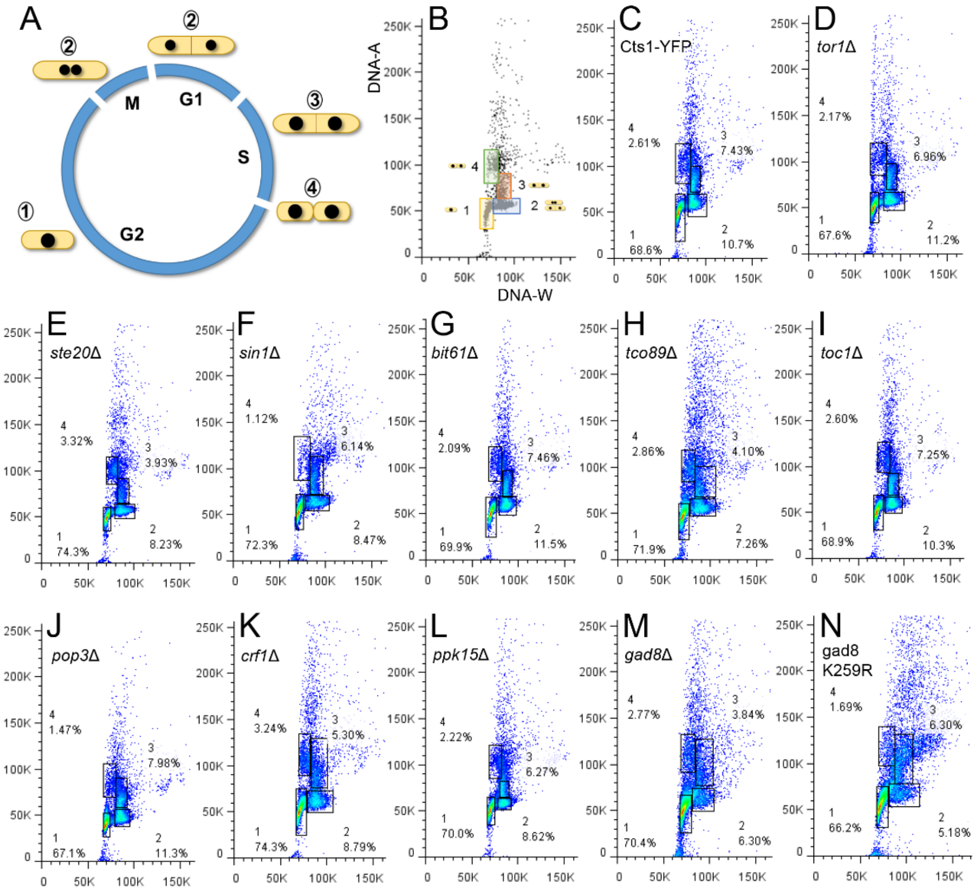

O

	wt	tor1Δ	*ste20Δ*	*sin1Δ*	*bit61Δ*	*tco89Δ*	*toc1Δ*	*pop3Δ*	*crf1Δ*	*ppk15Δ*	*gad8Δ*	gad8 K259R
1	68.6 ±2.7%	67.6 ±2.6%	74.3 ±2.55%	72.3 ±2.85%	69.9 ±2.7%	71.9 ±2.75%	68.9 ±2.65%	67.1 ±3.8%	74.3 ±2.7%	70.0 ±3.25%	70.4 ±2.55%	66.2 ±2.7%
2	10.7 ±1.1%	11.2 ±1.3%	8.23 ±1.05%	8.47 ±1.35%	11.5 ±1.2%	7.26 ±1.5%	10.3 ±1.3%	11.3 ±1.1%	8.79 ±1.02%	8.62 ±1.06%	6.30 ±1.16%	5.18 ±1.29%
3	7.43 ±0.64%	6.96 ±0.51%	3.93 ±0.58%	6.14 ±0.79%	7.46 ±0.99%	4.10 ±0.59%	7.25 ±0.95%	7.98 ±0.99%	5.3 ±0.66%	6.27 ±0.63%	3.84 ±0.54%	6.3 ±0.58%
4	2.61 ±0.23%	2.17 ±0.36%	3.32 ±0.57%	1.12 ±0.22%	2.09 ±0.59%	2.86 ±0.81%	2.60 ±0.22%	1.47 ±0.27%	3.24 ±0.31%	2.22 ±0.37%	2.77 ±0.54%	1.69 ±0.24%

Figure S2. Cell cycle analyses of TOR mutants. (A) Schematic representation of fission yeast cells, showing their DNA content and size/shape along the different phases of the cell cycle [adapted from (89)]. (B) As previously shown (89), asynchronous, exponentially growing fission yeast cells can be separated into four subpopulations in a two-parametric DNA-W/DNA-A diagram obtained after flow cytometry (see Materials and Methods). Subpopulation 1 includes G2-cells with a single nucleus and 2C DNA content, subpopulation 2 includes G1- or late mitotic cells that also have 2C DNA content, subpopulation 3 includes cells in S phase with DNA content between 2C and 4C, while subpopulation 4 accounts for cell doublets, which have a very broad distribution on an FSC/SSC cytogram, in contrast to subpopulations 1-3, which are very well defined (89). (C-N) Exponentially growing cells of Cts1-YFP background (control and TOR knockout mutants, as indicated) were treated with Sytox Green fluorescent DNA dye, before continuing to flow cytometry. DNA-A/DNA-W diagrams are presented showing the average values of cells in each of the four subpopulations after two independent repeats of the experiment. (O) The average values of the cells in each cell subpopulation is presented along with the ± standard deviation values, as calculated after two independent repeats.

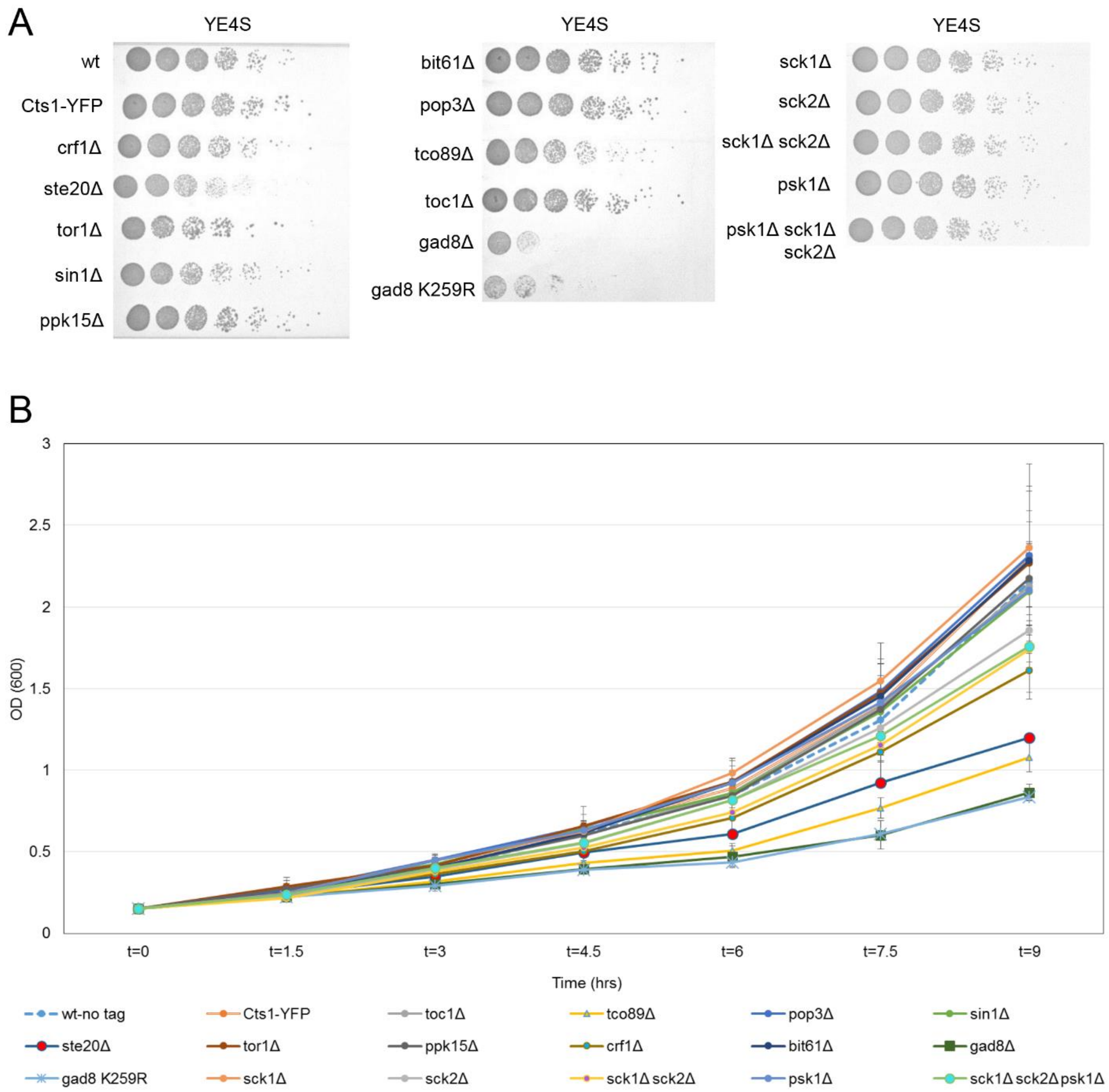

Figure S3. Growth rate analyses of TOR mutants. (A) TOR mutants of Cts1-YFP background, along with control strains (wild type and Cts1-YFP) were grown in YE4S until reaching an OD_{600}~1. Seven serial dilutions (1/5) were spotted on YE4S agar plates. Photos of the plates were taken after 2 days of incubation at 30^0C. (B) Early log phase cells of Cts1-YFP background, along with TOR mutant strains were cultured in YE4S, and growth was monitored for a period of 9 hours. The experiment was repeated in triplicate and error bars show the mean ± standard deviation as calculated after three biological repeats. The slower growth is significant for *gad8*Δ and gad8 K259R strains ($P<0.05$), as compared to the wild type growth.
